# Characterization of *Bacillus velezensis* UTB96, Demonstrating Improved Lipopeptide Production Compared to the Strain *B. velezensis* FZB42

**DOI:** 10.3390/microorganisms10112225

**Published:** 2022-11-10

**Authors:** Maliheh Vahidinasab, Isabel Adiek, Behnoush Hosseini, Stephen Olusanmi Akintayo, Bahar Abrishamchi, Jens Pfannstiel, Marius Henkel, Lars Lilge, Ralf T. Voegele, Rudolf Hausmann

**Affiliations:** 1 Department of Bioprocess Engineering (150k), Institute of Food Science and Biotechnology, University of Hohenheim, Fruwirthstraße 12, 70599 Stuttgart, Germany; 2 Department of Phytopathology (360a), Institute of Phytomedicine, Faculty of Agricultural Sciences, University of Hohenheim, Otto-Sander-Str. 5, 70599 Stuttgart, Germany; 3 Core Facility Hohenheim, Mass Spectrometry Unit, University of Hohenheim, August-von-Hartmann-Str. 3, 70599 Stuttgart, Germany; 4 Cellular Agriculture, TUM School of Life Science, Technical University of Munich, Gregor-Mendel-Str. 4, 85354 Freising, Germany

**Keywords:** *Bacillus*, surfactin, fengycin, iturin A, bacillomycin D, lipopeptide, secondary metabolite, antimicrobial, biosurfactant, fungicide

## Abstract

*Bacillus* strains can produce various lipopeptides, known for their antifungal properties. This makes them attractive metabolites for applications in agriculture. Therefore, identification of productive wild-type strains is essential for the development of biopesticides. *Bacillus velezensis* FZB42 is a well-established strain for biocontrol of plant pathogens in agriculture. Here, we characterized an alternative strain, *B. velezensis* UTB96, that can produce higher amounts of all three major lipopeptide families, namely surfactin, fengycin, and iturin. UTB96 produces iturin A. Furthermore, UTB96 showed superior antifungal activity towards the soybean fungal pathogen *Diaporthe longicolla* compared to FZB42. Moreover, the additional provision of different amino acids for lipopeptide production in UTB96 was investigated. Lysine and alanine had stimulatory effects on the production of all three lipopeptide families, while supplementation of leucine, valine and isoleucine decreased the lipopeptide bioproduction. Using a 45-litre bioreactor system for upscaling in batch culture, lipopeptide titers of about 140 mg/L surfactin, 620 mg/L iturin A, and 45 mg/L fengycin were achieved. In conclusion, it becomes clear that *B. velezensis* UTB96 is a promising strain for further research application in the field of agricultural biological controls of fungal diseases.

## 1. Introduction

*Bacillus velezensis* is known to be associated with plant roots. It is indigenous to the rhizosphere and provides many benefits to the plants [1,2]. Its value for agronomic applications lies primarily in the production of a variety of secondary metabolites that can benefit the plant by acting directly as antagonists against fungal pathogens or indirectly as triggers for systemic plant resistance, and by retaining nutrients such as nitrogen, phosphate, and iron that can promote plant growth. Therefore, *Bacillus velezensis* is recognized as a plant-growth-promoting rhizobacterium (PGPR) [3,4,5,6,7,8].

**Figure 1:**
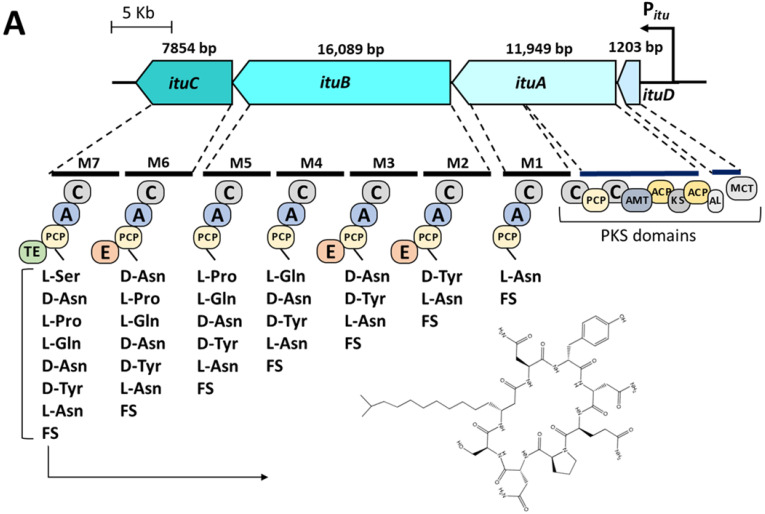

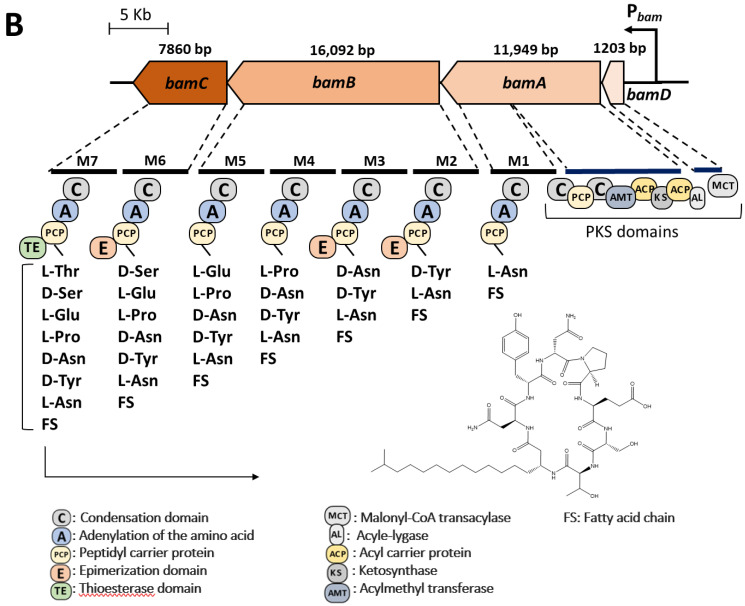
Genomic organization and modulation of the NRPSs producing bacillomycin D and iturin A. (**A**) Iturin A operon in *B. velezensis* UTB96 contains four ORFs of *ituD*, *ituA*, *ituB*, and *ituC* (37,246 bp), (**B**) Bacillomycin D operon in *B. velezensis* FZB42 contains four ORFs of *bamD, bamA, bamB*, and *bamC* (37,251 bp) [17]. The organization of the modules is comparable in both operons, although modules 4–7 catalyze different amino acids for the integration into the peptide moiety.

Among the *Bacillus* secondary metabolites, one of the most important groups are lipopeptides, represented by surfactin, iturin, and fengycin [9]. Notably, *Bacillus velezensis* wild-type strains can produce all three types of lipopeptides [10]. Lipopeptides are synthetized by complex non-ribosomal peptide synthetases (NRPSs), hybrid polyketide synthases (PKSs) or a combination of both enzymes [11,12]. These multi enzyme complexes are encoded in huge operons and work as a ribosomal-independent “machine” for the biosynthesis of respective lipopeptides. The structure of lipopeptides consists of a cyclic peptide chain of 7 to 10 amino acids linked to a β-amino or β-hydroxy fatty acid residue containing 14 to 19 carbons. The surfactin family consists of a hepta-peptide and the fengycin family consists of a deca-peptide attached to a β-hydroxy fatty acid chain with variable chain length [13,14]. All iturins consist of a hepta-peptide linked to a ß-amino fatty acid often of *iso*- or *anteiso*-type and of variable length (C_14_-C_18_) [15] (Figure 1). Moreover, differences in amino acids and fatty acid residues lead to various homologs [16].

Several significant biological functions have been reported for these three lipopeptide families, such as antifungal, antibacterial, antiviral, antioxidant, and antitumor activities [18,19,20,21]. Nevertheless, there is the suspicion that many of the described effects become effective only through the interaction of the lipopeptides with other bioactive metabolites [22]. Regardless, lipopeptides have the potential to find application in a variety of areas [19,23]. In more detail, surfactins are better known as biosurfactant metabolites and iturins reveal the strongest antifungal activity followed by fengycins [9,24]. Many studies have shown that the antifungal activity of fengycins is mostly limited to filamentous fungi [25], while iturins have a broader range and even stronger antifungal activity. Iturins have direct antagonistic activity and can destroy the fungal cell membrane through a variety of mechanisms [23]. Based on the unique structure of each lipopeptide, the respective molecule exhibits a special structure-based property. Interestingly, even within the variants of one lipopeptide, there could be a different antifungal activity. It has been already shown that iturins with longer fatty acid chains have stronger antimicrobial activity [26,27,28]. However, although the bioactivity of lipopeptides, especially the iturin and fengycin families, is beneficial, it has not yet been possible to carry out targeted application trials with the isolated substance in agriculture or other disciplines. This is mainly due to the low lipopeptide titers synthesized by native wild-type producer strains, and thus costly and ineffective production. Most of biocontrol agents currently in use are based on living microorganisms, mostly as liquid suspensions and dried formulations prepared from durable spores [17]. As the native lipopeptide production capabilities seem to be insufficient, improved bacterial production strains and processes need to be developed. One approach for increased lipopeptide production is the enhanced availability of precursors, such as amino acids [29,30,31,32].

In the present study, to compare the wild-type strain *B. velezensis* UTB96 [33,34,35] with the established *B. velezensis* model strain FZB42 [36,37], the lipopeptide production was characterized. Differences in the operons encoding the lipopeptide-producing NRPSs were identified by bioinformatic analyses and the lipopeptide production validated by mass spectrometry analyses. In addition, the potential of UTB96 as a strain with antifungal properties was highlighted by comparative batch cultivation processes and antifungal approaches. In addition, strain UTB96 was applied in upscaling with 45-litre bioreactor systems and the lipopeptide production was analyzed, showing the potential of this strain for future studies in agriculture. Finally, to identify bottlenecks in the lipopeptide biosynthesis of UTB96, the influence of the availability of different amino acids on lipopeptide production was analyzed.

## 2. Materials and Methods

### 2.1. Bacterial and Fungal Strains

*Bacillus velezensis* strains UTB96 and FZB42 were used for characterization of lipopeptide production and their potential for antifungal activity. UTB96 was previously isolated from the soil around a pistachio tree in Iran [33] and is deposited at the Leibniz Institute German Collection of Microorganisms and Cell Cultures GmbH (DSMZ) under the accession number DSM 114406. *Bacillus velezensis* FZB42 (DSM 23117) is a model strain for Gram-positive PGPR and its antifungal activity has frequently been reported [20].

The fungal pathogen *Diaporthe longicolla* is described as a seedborne pathogen of soybean [38]. In this study, *D. longicolla* DPC_HOH20 causing pod and stem blight on soybean plants was kindly provided by the Institute of Phytomedicine of the University of Hohenheim [39].

### 2.2. Primers and DNA Sequencing

The list of primers is shown in the Supplementary Material S1. Chromosomal DNA was purified with the ready to use kit innuPREP Bacteria DNA Kit (Analytik Jena AG, Jena, Germany) and used as a template for amplifying DNA fragments (Phusion High-Fidelity Polymerase #M0530S, New England BioLabs, Frankfurt am Main, Germany) using a PCR thermal cycler (prqSTAR 96X VWR GmbH, Darmstadt, Germany). The amplified DNA fragments were purified using the QIAquick PCR and Gel Cleanup Kit (Qiagen, Hilden, Germany), according to the manufacturers’ instructions. All PCR fragments were sequenced by Eurofins Genomics Germany GmbH (Ebersberg, Germany). Finally, the DNA sequence was analyzed using the AntiSMASH database version 6 [40] and was blasted using the National Center for Biotechnology Information Blast database (Rockville Pike Bethesda, MD, USA).

### 2.3. LC-MS/MS Analyses of Lipopeptides

The LC-MS/MS analysis of lipopeptides was performed on a 1290 UHPLC system (Agilent, Waldbronn, Germany) coupled to a Q-Exactive Plus Orbitrap mass spectrometer equipped with a heated electrospray ionization (HESI) source (Thermo Fisher Scientific, Bremen, Germany). Analyte separation was achieved by an ACQUITY CSH C18 column (1.7 μm, 2.1 μm × 150 mm, Waters, Eschborn, Germany). The column temperature was maintained at 40 °C. Samples were dissolved in methanol and 10 µL of each sample was injected. Mobile phase A was 0.2% formic acid in water, and mobile phase B 0.2% formic acid in acetonitrile. A constant flow rate of 0.3 mL/min was used and the gradient elution was performed as follows: 40–70% B from 0 to 12 min, 70–95% B from 12 to 20 min, isocratic at 95% B from 20 to 24 min, the system was returned to initial conditions from 95% B to 40% B from 24 to 26 min.

The HESI source was operated in the positive ion mode with a spray voltage of 4.20 kV and an ion transfer capillary temperature of 360 °C. The sweep gas and auxiliary pressure rates were set to 60 and 20, respectively. The S-Lens RF level was 50%, and the auxiliary gas heater temperature was 150 °C. The Q-Exactive Plus mass spectrometer was calibrated externally in positive ion mode using the manufacturers calibration solutions (Pierce/Thermo Fisher, Germany). Mass spectra were acquired within the mass range of 500 to 1600 *m*/*z* at a resolution of 70,000 FWHM using an Automatic Gain Control (AGC) target of 1.0 × 10E6 of and 100 ms maximum ion injection time. Data dependent MS/MS spectra in a mass range of 50 to 1600 *m*/*z* were generated for the five most abundant precursor ions with a resolution of 17,500 FWHM using an AGC target of 3.0 × 10E6 and 100 ms maximum ion injection time and a stepped collision energy of 15, 30 and 45. The *m*/*z* values of iturin A, bacillomycin D, fengycin and surfactin lipopeptides were predefined in an inclusion list to ensure that MS/MS spectra of corresponding precursors were acquired. Xcalibur™ software version 4.3.73.11 (Thermo Fisher Scientific, San Jose, USA) was used for data acquisition and data analysis. Peak areas of individual lipopeptides were calculated based on extracted ion chromatograms (XICs) of the corresponding precursor ions. Samples were analyzed in triplicate. Assignment of individual lipopeptides was based on the precise *m*/*z* value of the precursor ion, manual inspection of corresponding MS/MS spectra and comparison with available MS/MS spectra from literature [41,42,43,44].

### 2.4. Media and Cultivation Procedure

#### 2.4.1. Shake Flask Cultivations

A mineral salt medium based on the fermentation medium of Vahidinasab et al. [45] was used for cultivation in shake flasks. The initial pH of the medium was set as 7 and it consisted of 4.0 × 10^−6^ M Na_2_EDTA × 2 H_2_O, 7.0 × 10^−6^ M CaCl_2_, 4.0 × 10^−6^ M FeSO_4_ × 7 H_2_O, 1.0 × 10^−6^ M MnSO_4_ × H_2_O, 50 mM Urea, 30 mM KH_2_PO_4_, 40 mM Na_2_HPO_4_ × 2 H_2_O and 8.0 × 10^−4^ M MgSO_4_ × 7 H_2_O. In addition, glucose was used as the sole carbon source at concentrations of 8 g/L, 20 g/L, or 40 g/L (*m*/*v*). Furthermore, to verify the impact of amino acid supplementation on lipopeptide formation, 0.5 mM of a single amino acid was optionally added to the cultivation process.

The first preculture was prepared by inoculating 10 mL of LB medium (10 g/L tryptone, 5 g/L NaCl, 5 g/L yeast extract) with 10 μL of a glycerol stock solution in a 100 mL baffled shake flask. After 8 h of cultivation, the first preculture was used to inoculate 10 mL mineral salt medium with an initial optical density (OD_600_) of 0.1 as the second preculture. The second preculture was incubated for 10 to 12 h. Exponentially growing cells from the second preculture were washed and used for inoculation of the main culture with a final volume of 100 mL and an initial OD_600_ of 0.1 in a 1 L baffled shake flask. All cultivations had three biological replicates and were performed at 30 °C and 120 rpm in an incubation shaker (Innova 44^®^R, Eppendorf AG, Hamburg, Germany).

#### 2.4.2. Bioreactor Cultivations

Batch-bioreactor cultivations were carried out with the two biological replicates in 42 L custom-built bioreactors (ZETA GmbH, Graz/Lieboch, Switzerland) with a filling volume of 20 kg. The media used for the fermentation processes were described by Willenbacher et al. [46].

The bioreactors are equipped with pH (EasyFerm Bio HB Arc 120, Hamilton Bonaduz AG, Bonaduz, Switzerland) and pO_2_ probes (VisiFerm DO ARC 120 H0, Hamilton Bonaduz AG), a temperature sensor and three Rushton turbines. The temperature was fixed at 30 °C, and the pH was regulated to a value of 7.0 by the addition of 4 M NaOH or 4 M H_3_PO_4_. At the beginning of the fermentation process, the stirrer was adjusted to constant 300 rpm; afterwards, the stirrer was regulated by the online control of the dissolved oxygen that was set to a minimum of 20%. The airflow was adjusted to 0.07 vvm. Foam fractionation methods are described by Klausmann et al. [47] including the use of a foam centrifuge as well as the antifoam agent Contraspum A4050 (Zschimmer and Schwarz GmbH, Lahnstein, Germany). Moreover, a foam trap was installed in front of the exhaust gas filter to collect the potentially over-foaming medium.

### 2.5. Lipopeptide Extraction and Quantitative Analysis

Cell-free supernatants were obtained after 10 minutes centrifugation at 4700 rpm and were used for extraction of lipopeptides according to the method described by Yazgan et al. [48]. Specifically, a volume of 2 mL of the cell-free supernatant was mixed three times with 1 mL of 1-butanol 95% (*v*/*v*) by vortexing for 1 min, followed by 5 min centrifugation at 3000 rpm. The organic phases were pooled and used for evaporation of butanol phases (RVC2-25 Cdplus, Martin Christ Gefriertrocknungsanlagen GmbH, Osterode am Harz, Germany) at 10 mbar and 60 °C. The remaining residues were dissolved in 2 mL methanol. To quantify the total amounts of lipopeptides, purified fengycin was purchased from Lipofabrik (Lesquin, France) and surfactin and iturin A standards were ordered from Sigma–Aldrich (Seelze, Germany). High-performance thin-layer chromatography (HPTLC) was performed for quantification of lipopeptides. All HPTLC instruments and chambers were from CAMAG (Muttenz, Switzerland) and instruments were controlled by winCATS Software 1.4.7 as described previously [49].

### 2.6. Evaluation of Antifungal Activity

Antagonism of *B. velezensis* UTB96 and *B. velezensis* FZB42, respectively, against *Diaporthe longicolla* DPC_HOH20 was determined using a dual-culture assay according to a method previously described by Johnson et al. [50]. Specifically, a 0.6 cm mycelial plug from the margins of an actively growing 5-days-old culture of *D. longicolla* was placed in the corner of the plate with 150 mm distance from the edge of the Petri dish containing potato dextrose agar (PDA) and LB agar (1:1) medium.

In a first approach, sterile filter paper (MN 617 G) with the thickness of 0.22 mm and diameter of 0.5 cm was soaked in a cell suspension of fresh bacterial overnight culture in LB medium with an OD_600_ of 2.5. The soaked filter paper was placed 150 mm from the edge of the Petri dish.

In a second approach, instead of inoculating the plate with bacterial cells, filter sterilized cell-free supernatant from *B. velezensis* UTB96 and *B. velezensis* FZB42, respectively, was used. The supernatant was taken from a cell suspension cultivated for 48 h in mineral salt medium with 40 g/L glucose. Finally, a volume of 200 µL of the cell-free supernatant was transferred to an 8 mm diameter well, which was 150 mm in distance from the edge of the Petri dish.

In the control treatment, a mycelial plug of *D. longicolla* was placed in the plate without bacterial strains. All plates were incubated in the dark at room temperature for 5 days. To measure the percentage of inhibition radial growth (PIRG), the following formula was used:

PIRG (%) = [(R1 − R2)/R1] × 100

where R1 represents the radius of the control fungus and R2 is the radius of the fungi in treatment with the bacteria. Each treatment was replicated three times and the experiment was repeated thrice.

### 2.7. Data Analysis

The growth rate μ, specific productivity (q*_P/X_*), product per substrate (Y*_P/S_*) and product per biomass (Y*_P/X_*) for each lipopeptide, as well as the yield of biomass per substrate (Y*_X/S_*), were determined using the equations shown in [13]. Variable Y*_X/S_*was quantified at the maximum cell dry weight (CDW*_max_*), while Y*_P/S_*, Y*_P/X_* and q*_P/X_* were quantified at the maximum lipopeptide concentrations. The statistical analyses, such as One-way ANOVA, were performed using SigmaPlot (version 13) software. In all the graphs, Error bars indicate the standard deviation between different sample replicates.

## 3. Results

### 3.1. Bioinformatic Analyses of Lipopeptide Biosynthesis

*Bacillus velezensis* has been described extensively for its bioproduction of lipopeptides and can be used in agriculture as a biofungicide [51]. Therefore, in this study, the capability of *B. velezensis* UTB96 for lipopeptide production was first analyzed by bioinformatic approaches, using the genome of the model strain *B. velezensis* FZB42 (accession number: NC_009725.2) as reference. Using the AntiSMASH tool version 6 software [40,52,53], three operons encoding nonribosomal peptide synthetases (NRPSs) were identified for both strains. Besides the commonly known NRPS for biosynthesis of surfactin and fengycin, another gene cluster was identified, which showed only moderate comparability to the *bam* operon of the FZB42 strain encoding for the biosynthesis of bacillomycin D (Table 1).

To determine the type of iturin produced by *B. velezensis* UTB96, the nucleotide sequence of the whole iturin operon was analyzed. The sequencing results revealed an operon length of 37,246 bp including four open reading frames (ORFs) encoding for iturin A biosynthesis in UTB96 (accession number: OK274217.1). A subsequent AntiSMASH analysis revealed the modulation of the NRPS (Figure 1A). In this way, seven amino acid-activating modules responsible for the biosynthesis of the peptide ring, as well as the modules for fatty acid maturation were identified. Compared to the bacillomycin D biosynthesis of FZB42 (Figure 1B), both iturin versions share the first three amino acids in the circular peptide ring (L-Asn, D-Tyr, L-Asn) linked to a β-amino fatty acid, while the next four amino acids are different.

### 3.2. Comparative Structure-Based Iturin A and Bacillomycin D Analysis by Mass Spectrometry

**Figure 2:**
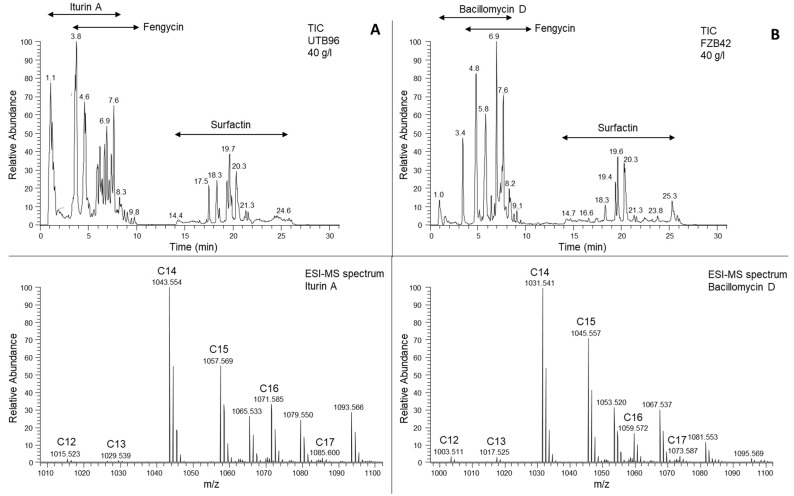
LC-ESI-MS analysis of the lipopeptide compounds produced by the *B. velezensis* UTB96 and *B. velezensis* FZB42 strains. (**A**) Total ion chromatogram (TIC, upper panel) and ESI-MS spectrum (lower panel) of the extracted iturin A lipopeptides from *B. velezensis* UTB96. The ESI-MS spectrum shows *m*/*z* values of protonated iturin A lipopeptides [M+H]^+^ eluted in the time interval from 0.8–8 min. Fatty acid chain length of different iturin A lipopeptides is indicated. (**B**) Total ion chromatogram (TIC, upper panel) and ESI-MS spectrum (lower panel) of the extracted bacillomycin D lipopeptides *B. velezensis* FZB42. The ESI-MS spectrum shows *m*/*z* values of protonated bacillomycin D lipopeptides [M+H]^+^ eluted in the time interval from 0.8–8 min. Fatty acid chain length of different bacillomycin D lipopeptides is indicated.

To further characterize the congener composition of the two types of iturins, as well as the surfactin and fengycin variants throughout the cultivation process, samples were taken after 48 h of cultivation and analyzed by liquid chromatography electrospray mass spectrometry (LC-ESI-MS/MS). The relative abundance of congeners was calculated using the corresponding peak areas. Both strains, FZB42 and UTB96, produced all three major classes of lipopeptides, namely iturin, fengycin, and surfactin. While *B. velezensis* FZB42 exclusively produced bacillomycin D, *B. velezensis* UTB96 exclusively produced iturin A lipopeptides (Figure 2). In *B. velezensis* FZB42, the peaks within the range of **m*/*z** = 989.488 to 1073.582 were assigned to protonated ion species [M+H]^+^ of bacillomycin D based on precise *m*/*z* values and the corresponding MS/MS spectra (data not shown). The C15 congener of bacillomycin D was the most abundant (approx. 45%) through the cultivation, followed by C14 (~35%). In contrast, C16 (~15%) and C17 congeners (~5%) were produced less during the cultivation period. In comparison, *B. velezensis* UTB96 produced iturin A congeners of C14, C15 and C16 at similar proportions (approx. 34, 33 and 30%, respectively) throughout the cultivation process, while C17 (~3%) was significantly underrepresented. The mass spectra of iturin A congeners were in the mass range of **m*/*z** = 1015.516 to 1085.594.

Fengycin produced by *B. velezensis* UTB96 and FZB42 is a mixture of several homologs based on the length of saturated or unsaturated fatty acid chain and variants within peptide moiety. For both strains, fengycin peaks with saturated fatty acid chain at **m*/*z** = 1435.766 to 1519.858 and for unsaturated fatty acid chain at **m*/*z** = 1433.787 to 1489.850 were observed. Mainly, fengycin A and B with saturated or unsaturated fatty acid chain with 14 to 18 carbon atoms for both strains were detected, with the saturated fatty acid chain variants being more abundant in both strains. In addition, several fengycin variants with substitutions in the peptide moiety were detected as described by Pathak et al. [44]. Both strains produced Fengycin A (Ala6, Ile10), Fengycin B (Val6, Ile10) as well as Fengycin A2 (Ala6, Val10) and Fengycin B2 (Val6, Val10).

Surfactin lipopeptides comprised a range of different surfactins congeners with saturated fatty acid chain between 12 to 17 carbon atoms that were detected in both strains at **m*/*z** = 994.636 to 1064.715. In the addition, several amino acid substitutions within the peptide sequence were observed as described by Kecskeméti et al. [42]. While the most abundant variant in both strains had the peptide sequence E-I/L-I/L-V-D-I/L- I/L [Sur], amino acid substitutions at positions 2, 4 and 7 (Val2, Val7, Ala4) as well as an aspartic acid 4-methyl ester at position 5 (AME5) were also detected (see extracted-ion chromatograms in Supplementary File S2).

### 3.3. Lipopeptide Production of B. velezensis UTB96 and FZB42 under Varying Substrate Availability

**Figure 3:**
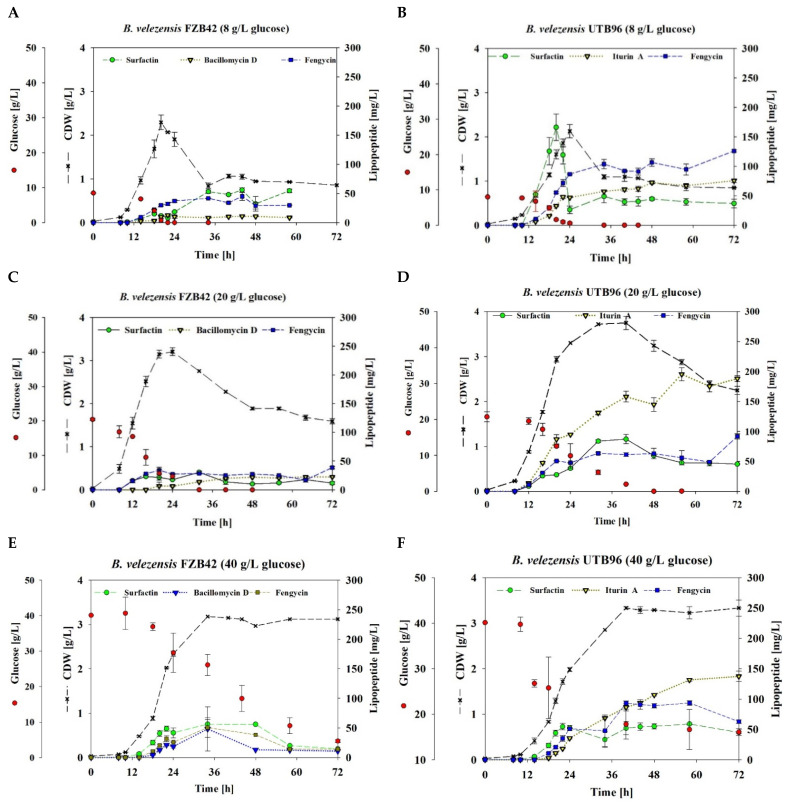
Overview of the time course of lipopeptide production by *B. velezensis* FZB42 and UTB96. The shake flask cultivations were conducted as biological triplicates in mineral salt medium containing different glucose concentration of 8 g/L (**A**,**B**), 20 g/L (**C**,**D**) and 40 g/L (**E**,**F**) at 30 °C, 120 rpm and initial pH of 7.

To get an overview of the production capability of *B. velezensis* UTB96, the extracellular accumulation of surfactin, fengycin and iturin was quantitatively analyzed and compared with the bioproduction of the model strain *B. velezensis* FZB42. Therefore, both strains were cultivated in shake flasks using mineral salt medium with different initial glucose concentrations of 8, 20 and 40 g/L. Figure 3 summarizes the surfactin, fengycin, and iturin A or bacillomycin D production, as well as the cell dry weight (CDW) and glucose consumption during a cultivation period of 72 h at 30 °C.

The comparison of growth behavior showed similar growth rates for both strains UTB96 and FZB42. However, a faster glucose consumption was detected for the FZB42 strain, resulting in slightly accelerated biomass formation compared to UTB96. After complete glucose depletion, a decline in CDW was detected for both *B. velezensis* strains.

With respect to the lipopeptide production, model strain FZB42 was able to synthesize stabilized titers of surfactin (~50 mg/L), fengycin (~30 mg/L) and bacillomycin D (~11 mg/L) using 8 g/L glucose. In comparison, the strain UTB96 produced clearly higher amounts of fengycin (~95 mg/L) and iturin A (~65 mg/L), while a maximum surfactin titer of 166 mg/L was reached during the exponential growth phase before a decline was detected and a final stabilized concentration of ~40 mg/L was maintained.

In cultivation with 20 g/L glucose, a higher availability of carbon source did not result in higher concentrations of fengycin and surfactin in FZB42 (~20 mg/L surfactin, ~30 mg/L fengycin) and UTB96 (~50 mg/L surfactin, ~90 mg/L fengycin), respectively, while for both iturin congeners, bacillomycin D and iturin A, increases of about two folds (22 mg/L) and three folds (190 mg/L) were observed. The highest initial glucose concentration used was 40 g/L. As a result, lipopeptide concentrations of 56.5 mg/L surfactin, 50.5 mg/L fengycin and 49 mg/L bacillomycin D were detected after 34 h of cultivation for FZB42 before a decline was observed for all lipopeptides. In contrast, relatively stable concentrations could be detected for UTB96 with the highest titers of 55 mg/L surfactin, 90 mg/L fengycin, and 100 mg/L iturin A in the stationary phase. However, a decline of surfactin and fengycin was detectable also in UTB96 at the end of cultivation. Overall, the UTB96 strain showed superior production values for all lipopeptide types (Table 2), especially for iturin and fengycin, which are more important for microbial antifungal activity [17].

### 3.4. Antifungal Activity

**Figure 4:**
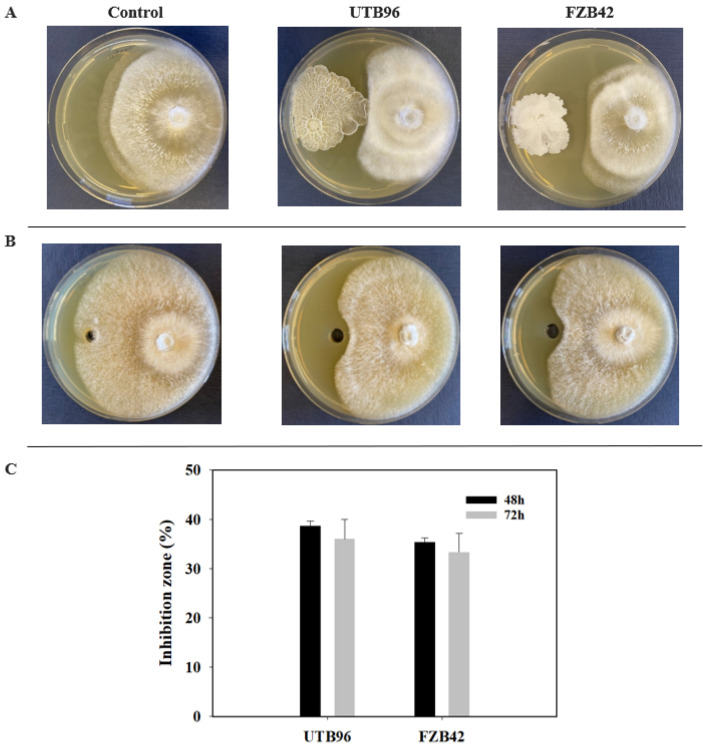
Comparison of antifungal activity; (**A**) cell suspension and (**B**) cell free supernatant taken after 48 and 72 h of *B. velezensis* strains UTB96 and FZB42 were each co-incubated with the soybean fungal pathogen *D. longicolla* strain DPC_HOH20 for 5 days. (**C**) The growth inhibitory effect of UTB96 and FZB42 against *D. longicolla* are summarized in bar graphs. Growth of *D. longicolla* DPC_HOH20 without the co-incubated *B. velezensis* strain was used as a control. Each treatment was repeated three times, while the approach was repeated thrice.

To characterize the antifungal properties of *B. velezensis* UTB96, inhibition assays of the cell suspension and the corresponding cell-free supernatant were analyzed and compared with the previously established strain FZB42 (Figure 4). For this purpose, a dual-culture assay was used to determine the growth inhibitory effect on the soybean fungal pathogen *Diaporthe longicolla* DPC_HOH20 as an indicator strain. In this way, distinct zones of inhibition were formed between the *D. longicolla* DPC_HOH20 and the bacterial strains, and the width of the inhibition zone remained unaffected for at least one month. In more detail, UTB96 was shown to have comparable antifungal activity in both cell-free supernatant and grown cells compared to the FZB42 reference strain. Specifically, UTB96 revealed approx. 28% (cell-mediated inhibition) and 9% (supernatant-mediated inhibition) larger zones of inhibition (Figure 4). However, both strains showed similar growth-inhibiting properties against *D. longicolla*, confirming the potential of strain UTB96 for agricultural applications.

### 3.5. Batch Bioreactor Cultivation

**Figure 5:**
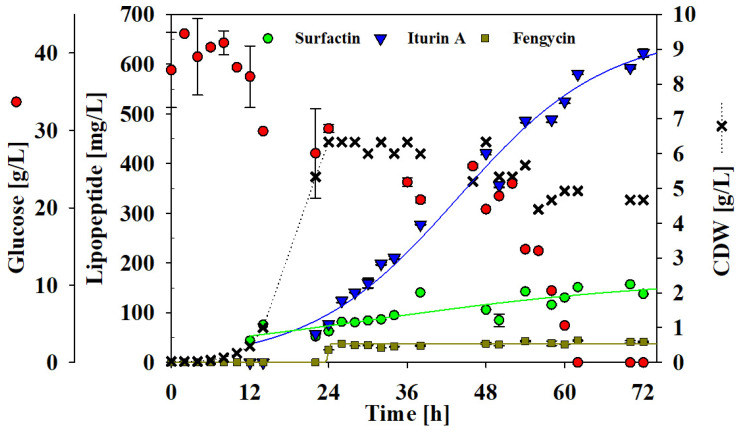
Time course of the cell dry mass (CDW) of the glucose and lipopeptide concentrations during a batch bioreactor fermentation with strain UTB96, glucose (red dots), CDW (black crosses), and the lipopeptide concentration of surfactin (green dots), iturin A (blue inverted triangles) and fengycin (brown squares).

As *B. velezensis* UTB96 appeared superior in the bioproduction of lipopeptides and showed slightly superior antifungal activity against the phytopathogen *D. longicolla*, a first attempt of upscaling with a batch culture to produce lipopeptides was made (Figure 5). For this purpose, a custom-built 42-L bioreactor with a filling volume of 20 kg medium was used. After inoculation, a lag phase of about 10 h occurred before an exponential growth phase started for the next 12 h. Afterwards, the cell culture entered the stationary phase and reached a maximum CDW of 6.3 g/L. The initial glucose concentration of 40 g/L was depleted after 62 h. In this way, a growth rate of 0.22 1/h and a biomass yield of 0.81 g/g was reached. Regarding lipopeptide production, surfactin was shown to be produced first after about 12 h and reached a maximum of 140 mg/L after 38 h. In contrast, an accumulation of iturin A started after about 22 h of cultivation. However, compared to surfactin and fengycin, a steady increase in the amount of iturin A was observed until the end of cultivation, with a maximum concentration of 620 mg/L. Finally, after 24 h of cultivation, fengycin production was detected, reaching a maximum titer of 42 mg/L, which remained constant until the end of the cultivation. Overall, improved production of surfactin (2.5-fold) and iturin A (3.2-fold) was observed, while the productivity of fengycin (2.2-fold) was reduced compared to previous shake flask cultivations. An overview of the lipopeptide production rates is provided in Table 2.

### 3.6. Effect of Amino Acid Availability on the Lipopeptide Production of B. velezensis UTB96

*Bacillus velezensis* UTB96 proved to be the comparatively more productive strain for lipopeptides and was therefore used for further studies on possible bottlenecks in amino acid precursor availability. To identify potential bottlenecks in the provision of amino acids as precursor molecules in the bioproduction of lipopeptides using the *B. velezensis* strain UTB96, a defined concentration of 0.5 mM of 21 different amino acids was supplemented to the previously described shake flask cultivation process with 40 g/L glucose. Because a stabilized lipopeptide concentration was previously ensured (Figure 3F), the effect of specific amino acid supplementation on the production of surfactin, fengycin and iturin A was analyzed after 48 h of cultivation. 

In the reference process without amino acid supplementation, strain UTB96 achieved a CDW of 3.65 g/L and concentrations of ~55 mg/L surfactin, ~107 mg/L iturin A, and ~89 mg/L fengycin. However, when histidine (−52%), aspartic acid (−52%), glutamic acid (−49%), glycine (−47%), arginine (−45%), methionine (−45%), lysine (−43%), glutamine (−41%), or alanine (−36%) were added, a drastically reduced biomass formation was observed. Nevertheless, the lipopeptide production was only slightly affected. Conversely, although the CDW was unaffected when leucine, valine or isoleucine were added, significantly reduced concentrations for all three lipopeptide families were detected. Thus, the addition of leucine reduced the amount of surfactin by 17% and of fengycin by 63%, while valine showed a negative effect on surfactin by 80% and on fengycin by 54%, and for isoleucine a reduction in surfactin by 74%, in iturin A by 41% and in fengycin by 65% was observed. Further amino acids that have a negative effect on surfactin were glutamic acid (−41%), phenylalanine and tryptophane (−51%). In contrast, supplementation of lysine showed a remarkable positive effect on the production of surfactin by 43% and iturin A by 65% and slightly increased the fengycin production by 27%. Furthermore, the addition of alanine allowed an improvement in the production of surfactin by 31%, of iturin A by 77%, and of fengycin by 47%. In addition, surfactin production was improved by the addition of proline and ornithine (~30%). Interestingly, except for isoleucine, an improved iturin A production was observed with all amino acids, suggesting that the provision of a nitrogen source may have a positive effect on iturin A bioproduction. Specifically, phenylalanine and tryptophan followed by tyrosine were the best-influenced amino acids on production of iturin A.

## 4. Discussion

*Bacillus velezensis* has been reported several times for its antifungal activity and thus for its potential for the use in agriculture [33,34,35]. In this context, most *B. velezensis* strains can synthesize all three types of lipopeptides, namely surfactin, fengycin, and iturin. Fengycin and iturin in particular are associated with bioactivity against fungi [17]. In this study, the strain *B. velezensis* UTB96 was compared with the reference strain FZB42. Although only marginal differences were found in the nucleotide sequences of the operons encoding the corresponding NRPSs, the protein sequences of the iturin synthetases of UTB96 and FZB42 differ drastically (Table 1). Thus, strain UTB96 can synthesize iturin A, while FZB42 produces bacillomycin D. As these molecules differ in the peptide structure (Figure 1), different antifungal properties could be assumed. Iturin A was reported to cause cell wall disappearance, membrane degeneration and hyphal fragmentation [54]. This makes strain UTB96 a promising candidate for future studies as a potent natural iturin-A producer. A subsequent comparison of lipopeptide productivity showed that UTB96 is clearly more productive than the reference strain FZB42 (Figure 3). In particular, the production of fengycin and iturin was superior, which makes the UTB96 strain interesting for further strain engineering work, as the natural productivity of the antifungal lipopeptides iturin and fengycin is relatively high. Future comparative analyses of the proteome and the metabolome may reveal the reason for the better lipopeptide production of UTB96 compared to FZB42, although both strains are closely related in genome [33]. As *B. velezensis* UTB96 has superior lipopeptide production, it was reasonable to observe enhanced antifungal activity against *D. longicolla* compared to FZB42 (Figure 4). Previous studies have demonstrated that *Bacillus velezensis* strains have a broad antagonism activity against several fungal phytopathogens such as soil-borne pathogens *Fusarium graminearum*, *F. solani*, *F. oxysporum*, *Rhizoctonia solani*, *Ralstonia solanacearum*, *Rosellinia necatrix* [55,56,57,58] and the common postharvest pathogens like *Botrytis cinerea*, *Penicillium digitatum* and *Monilinia fructicola* [59,60,61]. More specifically, it is reported that the lipopeptides bacillomycin D and fengycin produced by FZB42 contribute significantly to antifungal activity [62,63]. Similarly, purified Iturin A is reported to be capable of suppressing *Fusarium* sp. at relatively low concentrations [64].

In the subsequent upscaling approach using a custom-built bioreactor system, *B. velezensis* UTB96 showed promising productivities (Figure 5). In particular, iturin A production did not show any plateau after the end of the cultivation process of 72 h. Consequently, UTB96 appears to be a potent strain for iturin A production reaching 620 mg/L iturin A at the end of batch bioreactor cultivation. To increase the iturin A production fed-batch cultivations with the appropriate feeding strategy is an obvious solution. Similar experiments on the time course and kinetics of iturin A production have already been performed by Jin et al. [65]. They applied a two-step glucose feeding strategy and were thus able to maintain glucose levels at a low concentration and a desirable ratio of spores to total cells. As a result, they were able to achieve twice the production of iturin-A (1.12 g/L) compared to batch fermentation.

**Figure 6:**
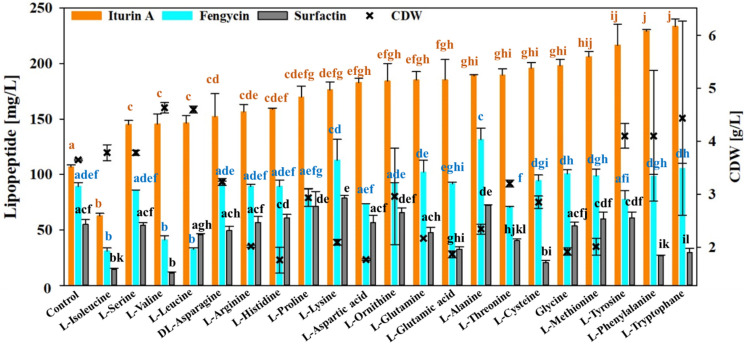
Lipopeptide concentrations and cell dry weight (CDW) achieved in dependence of different amino acid supplements (0.5 mM) by *B. velezensis* UTB96 after 48 h of cultivation. All tests were performed by On-Way ANOVA based on Holm–Sidak method. Bars with the same superscript letter are not significantly different (*p* < 0.05).

To gain further insights into possible bottlenecks in the bioproduction of lipopeptides in *B. velezensis* UTB96, different amino acids were added to the cultivation process (Figure 6). In this study, it was shown that the addition of the branched-chain amino acids valine, leucine, and isoleucine had a negative effect on the production of lipopeptides in the UTB96 strain. Wu et al. [30] reported that the production of iturin A by *Bacillus amyloliquefaciens* BPD1 was slightly increased by the addition of proline and asparagine, while the addition of serine significantly increased the yield of iturin A. These results contrast with a previous study, which reported that serine had no significant effect on iturin A production while asparagine had the best effect on the iturin A yield among other amino acids [32]. In the current study, the effect of serine supplementation on iturin A production by UTB96 was also not significant. This points to the possibility that each strain may need different types of amino acids as precursors for a specific lipopeptide production. The branched-chain amino acids valine, leucine, and isoleucine are essential amino acids [66]. Another explanation could be the action of global transcriptional regulator CodY, which is active in the presence of branched-chain amino acids. In this context, CodY acts as a repressor for the *srfA* operon expression [67], while a stimulatory effect has been described for the iturin operon (bacillomycin D) in the *B. amyloliquefaciens* fmbJ [68]; however, the possible effect of CodY on fengycin operon is unknown. Regarding the fengycin production, we have found that alanine is the most effective amino acid. Likewise, in the study by Yaseen et al. [31], alanine was shown to be the best nitrogen source among other amino acids to produce fengycin by *B. subtilis*. Indeed, in this study, we found that lysine and alanine had a stimulatory effect on the production of all three types of lipopeptides by UTB96. To determine the beneficial cellular adaptation in presence of these amino acids, future studies should focus on molecular adaptation in terms of the proteome and metabolome. For a deeper understanding, the omics profile of several bacterial strains can be compared. Notably, in this study, iturin A concentration increased independently of the amino acid supplemented, suggesting that higher availability of nitrogen sources stimulates iturin A bioproduction. All these findings should be taken into account in further studies, in which lipopeptide concentrations as well as spore number and quality should be simultaneously optimized in fed-batch bioreactor cultivations of *B. velezensis* UTB96, and the applicability of spore preparations as antifungal agents should be further elucidated in further application trials.

## Figures and Tables

**Table 1:** Comparison of the gene clusters encoding NRPS for biosynthesis of the main lipopeptides, surfactin, iturin, and fengycin, in the genomes of *Bacillus velezensis* UTB96 and the reference strain *Bacillus velezensis* FZB42.

		Similarity with the Reference Strain *Bacillus velezensis* FZB42	
Lipopeptide	Chromosomal Localization (from–to)	Similarity in Nucleotide Sequence	Similarity in Amino Acid Sequence
Surfactin	301,941 nt–366,339 nt	97.53%	91.67%
Iturin A	1,762,934 nt–1,800,180 nt	97.02%	67.94%
Fengycin	1,823,096 nt–1,860,765 nt	96.50%	97.08%

**Table 2:** Overview of the lipopeptide production parameters including surfactin, iturin A or bacillomycin D and fengycin by *B. velezensis* UTB96 and *B. velezensis* FZB42 and in shake flask cultivations with initial glucose concentrations of 8 g/L, 20 g/L and 40 g/L and in a batch bioreactor using 40 g/L of glucose.

				Surfactin			Iturin A in UTB96 or Bacillomycin D in FZB42			Fengycin		
***B. velezensis***	**Initial Glucose** **[g/L]**	**Y_X/S_** **[g/g]**	**Growth Rate** **µ [1/h]**	**Y_P/S_** **[mg/g]**	**Y_P/X_** **[mg/g]**	**q** **[mg/g.h]**	**Y_P/S_** **[mg/g]**	**Y_P/X_** **[mg/g]**	**q** **[mg/g.h]**	**Y_P/S_** **[mg/g]**	**Y_P/X_** **[mg/g]**	**q** **[mg/g.h]**
**UTB96**	**8 (SF)**	0.2 ± 0.01	0.2 ± 0.0	12.9 ± 1.16	27.4 ± 1.59	2.3 ± 0.13	5.8 ± 0.25	168.3 ± 8.76	1.2 ± 0.06	9.6 ± 0.13	281.5 ± 6.35	2.0 ± 0.04
	**20 (SF)**	0.6 ± 0.02	0.1 ± 0.0	3.6 ± 0.08	46.3 ± 5.93	0.6 ± 0.07	7.0 ± 0.86	134.4 ± 10.33	1.2 ± 0.09	3.4 ± 0.08	82.6 ± 1.41	0.7 ± 0.01
	**40 (SF)**	0.1 ± 0.01	0.1 ± 0.0	1.7 ± 0.1	62.4 ± 4.08	1.4 ± 0.09	3.5 ± 0.30	81.9 ± 9.51	0.6 ± 0.06	2.5 ± 0.13	55.4 ± 1.57	0,7 ± 0.01
	**40 (BR)**	0.6 ± 0.0	0.2 ± 0.0	8.4 ± 0.2	43.8 ± 3.8	0.9 ± 0.1	16.3 ± 0.8	249.4 ± 15.3	1.8 ± 0.0	1.2 ± 0.0	17.4 ±0.3	0.1 ± 0.0
**FZB42**	**8 (SF)**	0,2 ± 0.021	0.2 ± 0.00	5.0 ±0.24	120.6 ± 1.75	1.8 ± 0.00	1.4 ± 0	12.0 ± 2.04	0.3 ± 0.04	3.7 ± 0.59	81.9 ± 8.09	0.9 ±0.03
	**20 (SF)**	0.2 0.01	0.2 ± 0.01	1.2 ± 0.11	21.7 ± 1.68	0.3 ± 0.00	0.9 ± 0.01	35.2 ± 3.11	0.2 ± 0.02	1.3 ± 0.06	54.4 ± 6.23	0,3 ± 0.04
	**40 (SF)**	0.1 ± 0.0	0.1 ± 0.0	2.1 ± 0.04	39.6 ± 5.01	0.5 ± 0.06	0.4 ± 0.00	7.5 ± 0.53	0.1 ± 0.0	1.9 ± 0.4	32.1 ± 9.63	0.5 ± 0.14

SF—shake flask; BR—bioreactor.

## Data Availability

All raw data and biological material are saved in the institute of Food Science and Biotechnology, Department of Bioprocess Engineering (150 k), University of Hohenheim, Fruwirthstraße 12, Stuttgart 70599, Germany. In case of requirement, please contact the corresponding author with any detailed questions.

## Supplementary Materials

The following supporting information can be downloaded at: https://www.mdpi.com/article/10.3390/microorganisms10112225/s1, Supplementary Material 1: List of primers used in this study for amplification of iturin A operon in *Bacillus velezensis* UTB96. Supplementary Material 2: Extracted Ion Chromatograms for iturin A, surfactin and fengycin families produced by *B. velezensis* UTB96 as well as for bacillomycin D, surfactin and fengycin produced by *B. velezensis* FZB42. Assignments of lipopeptides were based on precise *m*/*z* values and manual inspection of the corresponding MS/MS spectra.
