# Supplementary material for: Characterization of Bacillus velezensis UTB96, Demonstrating Improved Lipopeptide Production Compared to the Strain B. velezensis FZB42
Source: Microorganisms. 2022 Nov 10;10(11):2225. doi: 10.3390/microorganisms10112225 (PMC9693074; doi:10.3390/microorganisms10112225)
Supplement: Supplementary file 1 [file microorganisms-10-02225-s001.zip › Supplementary Material 1.pdf]

Supplementary Material S1

Characterization of *Bacillus velezensis* UTB96, Demonstrating Improved Lipopeptide Production Compared to the Strain *B. velezensis* FZB42

Maliheh Vahidinasab [1,*], Isabel Adiek [1], Behnoush Hosseini [2], Stephen Olusanmi Akintayo [1], Bahar Abrishamchi [1], Jens Pfannstiel [3], Marius Henkel [4], Lars Lilge [1], Ralf Voegele [2] and Rudolf Hausmann [1]

[1]Department of Bioprocess Engineering (150k), Institute of Food Science and Biotechnology, University of Hohenheim, Fruwirthstraße 12, Stuttgart 70599, Germany

[2]Department of Phytopathology (360a), Institute of Phytomedicine, Faculty of Agricultural Sciences, University of Hohenheim, Otto-Sander-Str. 5, 70599 Stuttgart, Germany

[3]Core Facility Hohenheim, Mass Spectrometry Unit, University of Hohenheim, August-von-Hartmann-Str. 3, 70599 Stuttgart, Germany

[4]Department of Cellular Agriculture, TUM School of Life Science, Technical University of Munich, Gregor-Mendel-Str. 4, 85354 Freising, Germany

*Corresponding author: vahidin@uni-hohenheim.de

Supplementary Material S1. List of primers used in this study for amplification of iturin A operon in *Bacillus velezensis* UTB96

Name	Sequence 5′—3′	Purpose
s1250 F	GCCGTCATACAATTGAATCAG	Sequencing of nucleotide gaps in the *ituB* gene from *B. velezensis* UTB96
s1194 R	ATACGGTGCAAATGGTTCAAC	
s1661 F	GTTTCCGTCAGGAAGGTAAC	
s1662 R	GAGATTGTGTCAGACGGTG	
s1689 F	GCTCCGGATCAATAGGAAG	
s1690 R	GTGGGGATCAGAGATCAC	
s1665 F	GCTGACGATAAGTCAGTGTC	
s1666 R	CAACCATTGAACAGCTCGC	
s1201 F	CCGCTTCTTTGACAGCATC	Sequencing of nucleotide gaps in the *ituC* gene from *B. velezensis* UTB96
s1255 R	CGATATATCGAATGGCTGG	
s1254 F	CCAGCCATTCGATATATCG	
s1200 R	CTGGGATTGTTTCAATGTCAG	
s1659 F	CGGCCAGTATTTTTTCCAG	
s1660 R	TGGTTCTCCTGGTCGGAAT	
