# Supplementary material for: Characterization of Bacillus velezensis UTB96, Demonstrating Improved Lipopeptide Production Compared to the Strain B. velezensis FZB42
Source: Microorganisms. 2022 Nov 10;10(11):2225. doi: 10.3390/microorganisms10112225 (PMC9693074; doi:10.3390/microorganisms10112225)
Supplement: Supplementary file 1 [file microorganisms-10-02225-s001.zip › Supplementary Material 2.pptx]

## Slide 1
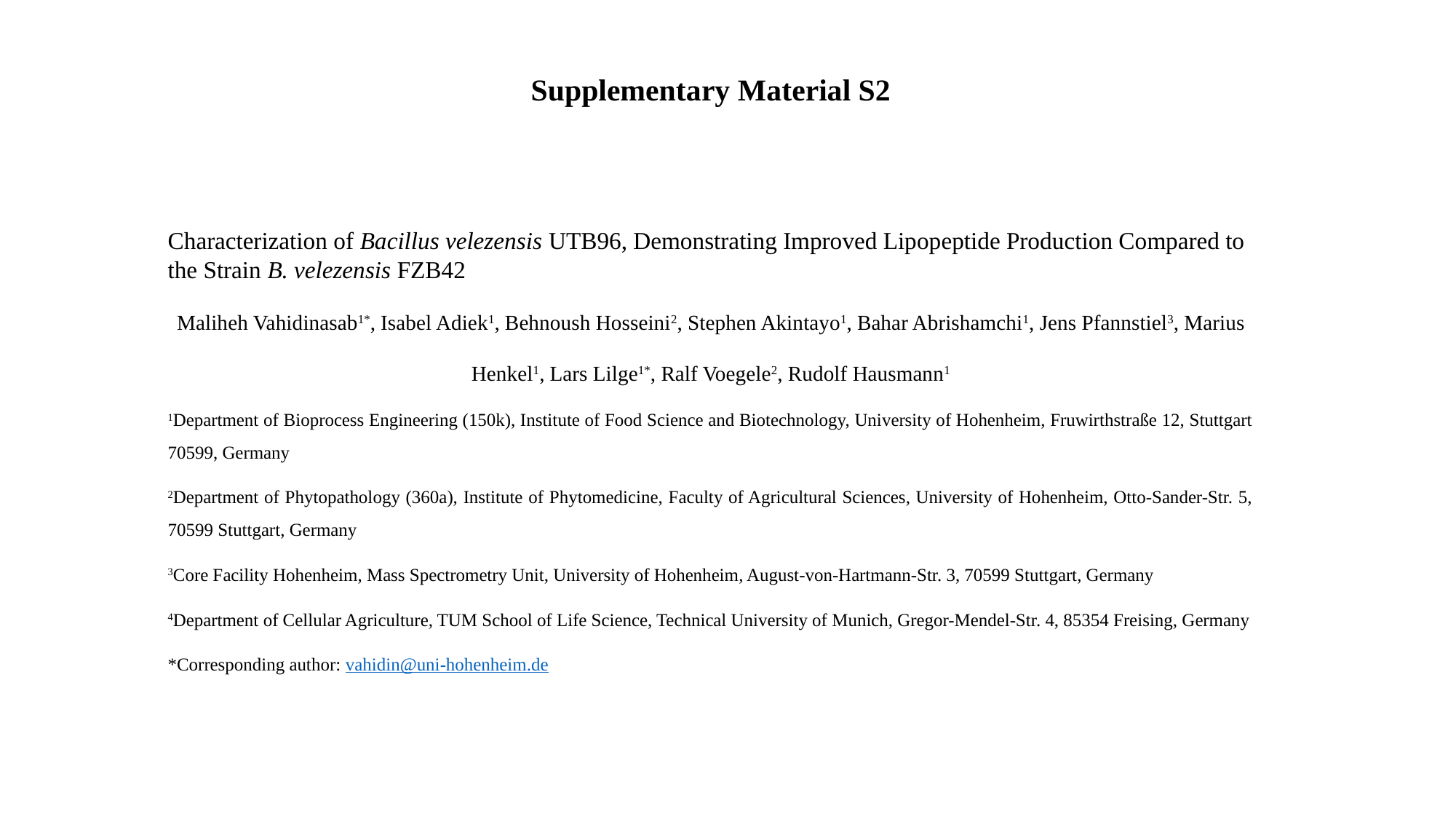

Supplementary Material S2
Characterization of Bacillus velezensis UTB96, Demonstrating Improved Lipopeptide Production Compared to the Strain B. velezensis FZB42
Maliheh Vahidinasab1*, Isabel Adiek1, Behnoush Hosseini2, Stephen Akintayo1, Bahar Abrishamchi1, Jens Pfannstiel3, Marius Henkel1, Lars Lilge1*, Ralf Voegele2, Rudolf Hausmann1
1Department of Bioprocess Engineering (150k), Institute of Food Science and Biotechnology, University of Hohenheim, Fruwirthstraße 12, Stuttgart 70599, Germany
2Department of Phytopathology (360a), Institute of Phytomedicine, Faculty of Agricultural Sciences, University of Hohenheim, Otto-Sander-Str. 5, 70599 Stuttgart, Germany
3Core Facility Hohenheim, Mass Spectrometry Unit, University of Hohenheim, August-von-Hartmann-Str. 3, 70599 Stuttgart, Germany
4Department of Cellular Agriculture, TUM School of Life Science, Technical University of Munich, Gregor-Mendel-Str. 4, 85354 Freising, Germany
*Corresponding author: vahidin@uni-hohenheim.de

## Slide 2
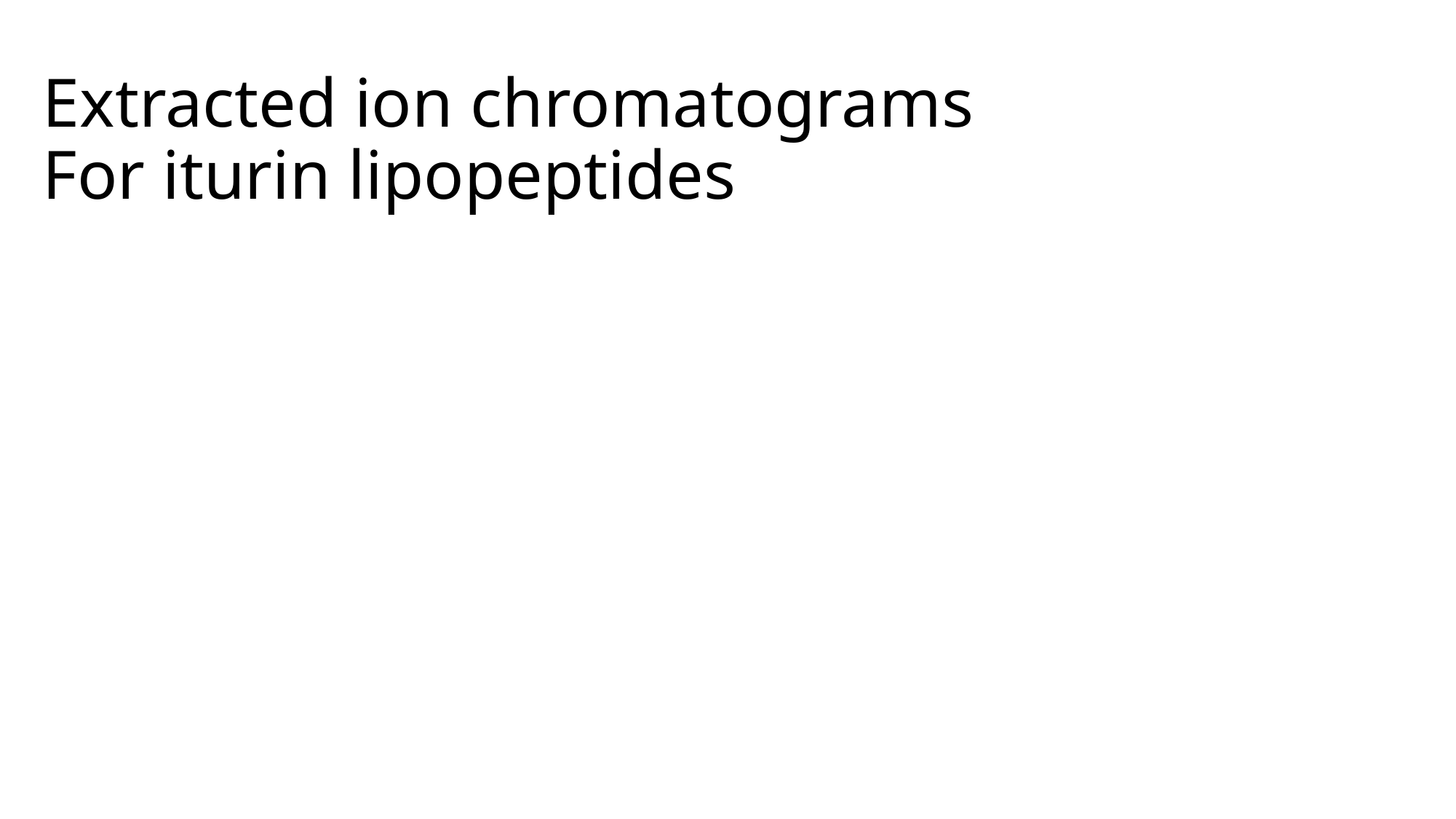

# Extracted ion chromatograms For iturin lipopeptides

## Slide 3
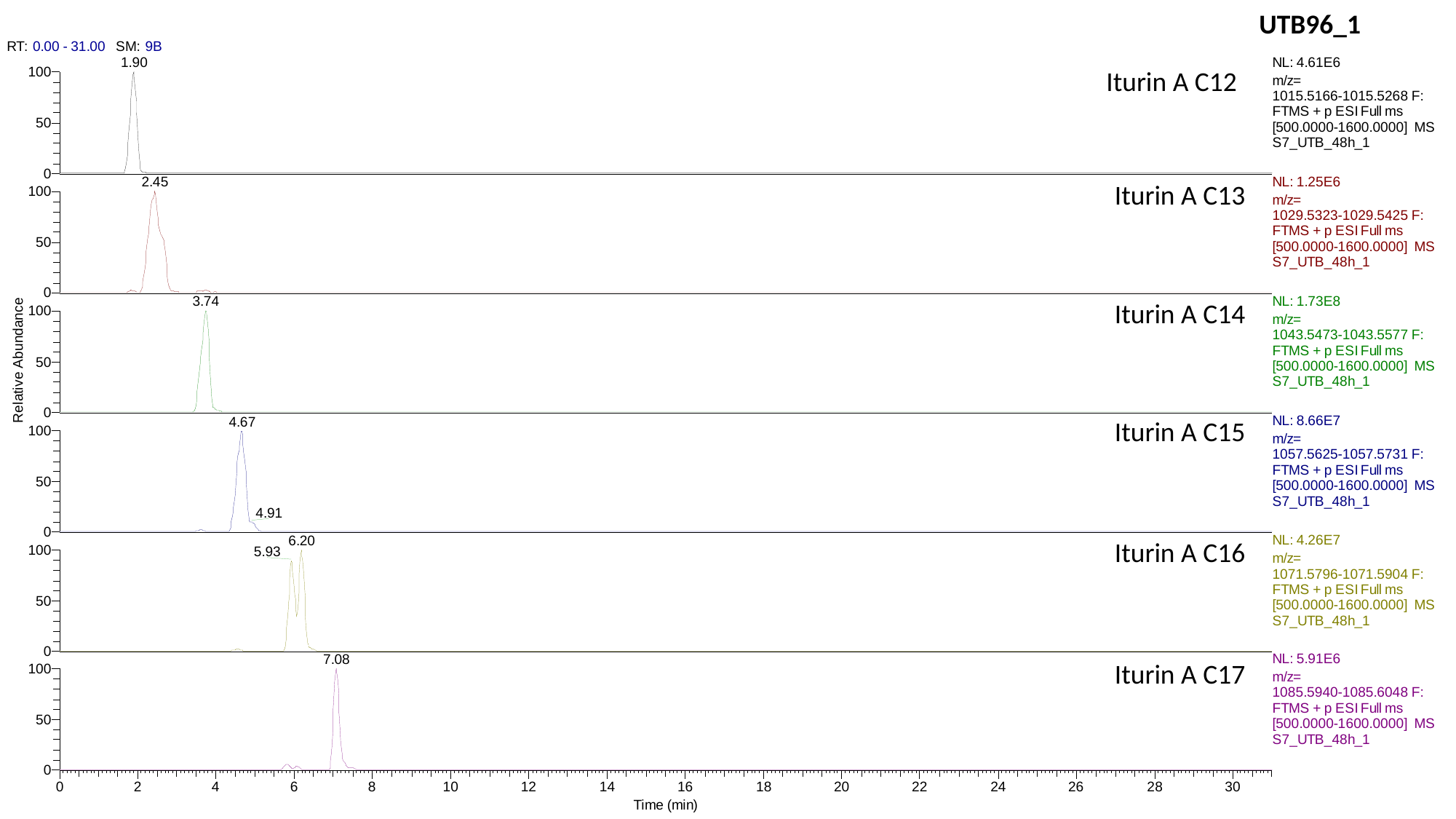

UTB96_1
Iturin A C12
Iturin A C13
Iturin A C14
Iturin A C15
Iturin A C16
Iturin A C17

## Slide 4
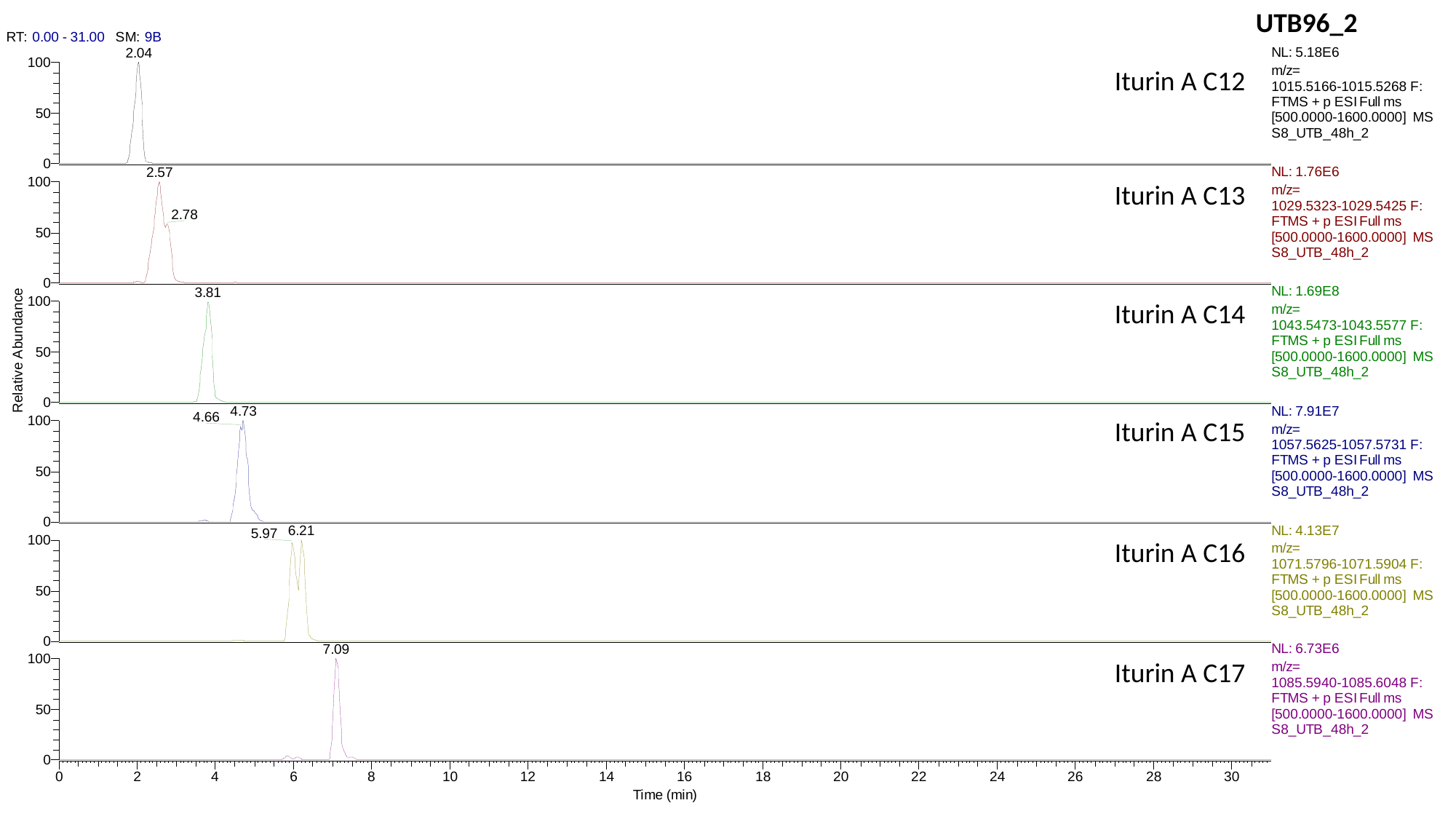

UTB96_2
Iturin A C12
Iturin A C13
Iturin A C14
Iturin A C15
Iturin A C16
Iturin A C17

## Slide 5
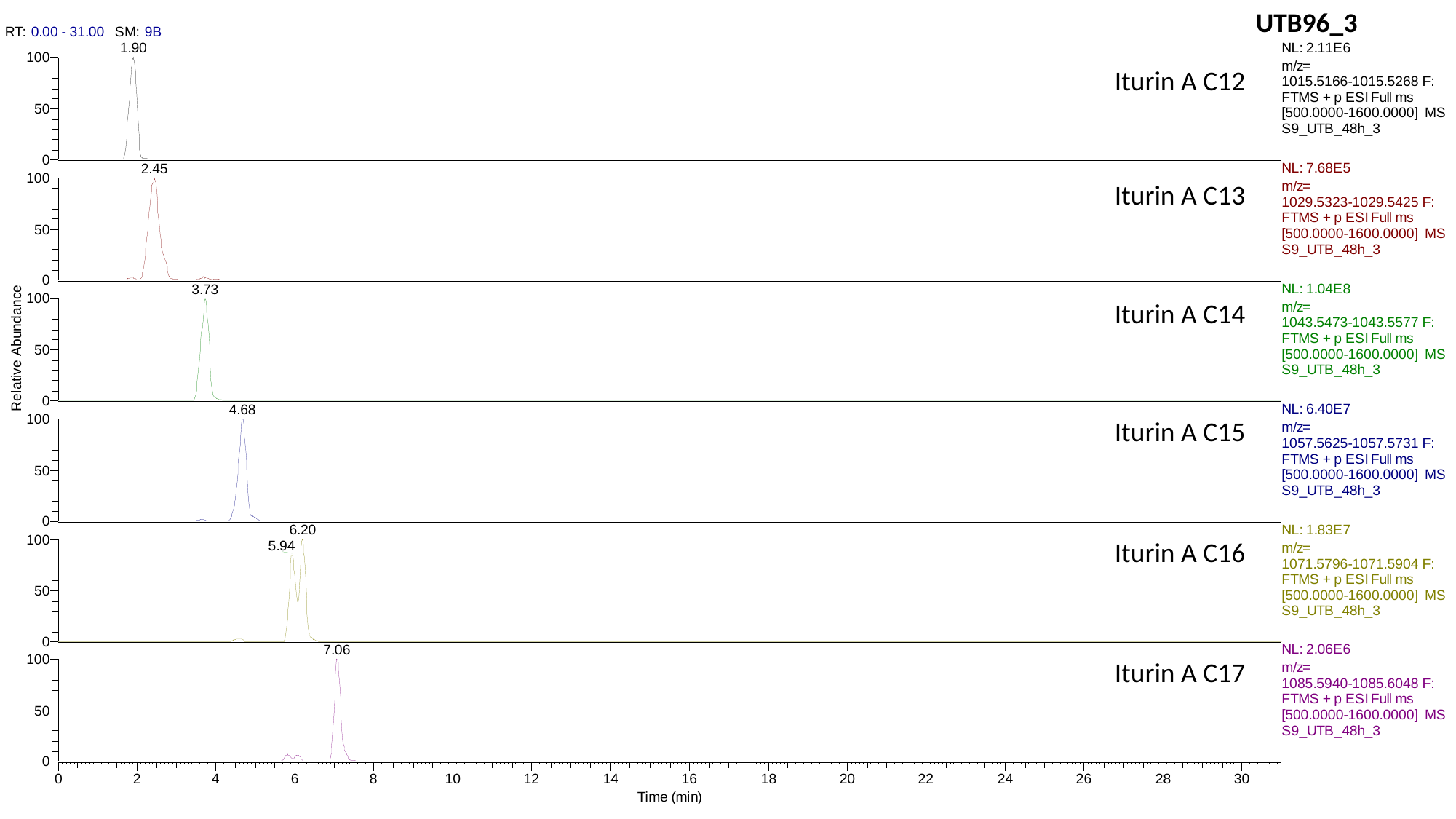

UTB96_3
Iturin A C12
Iturin A C13
Iturin A C14
Iturin A C15
Iturin A C16
Iturin A C17

## Slide 6
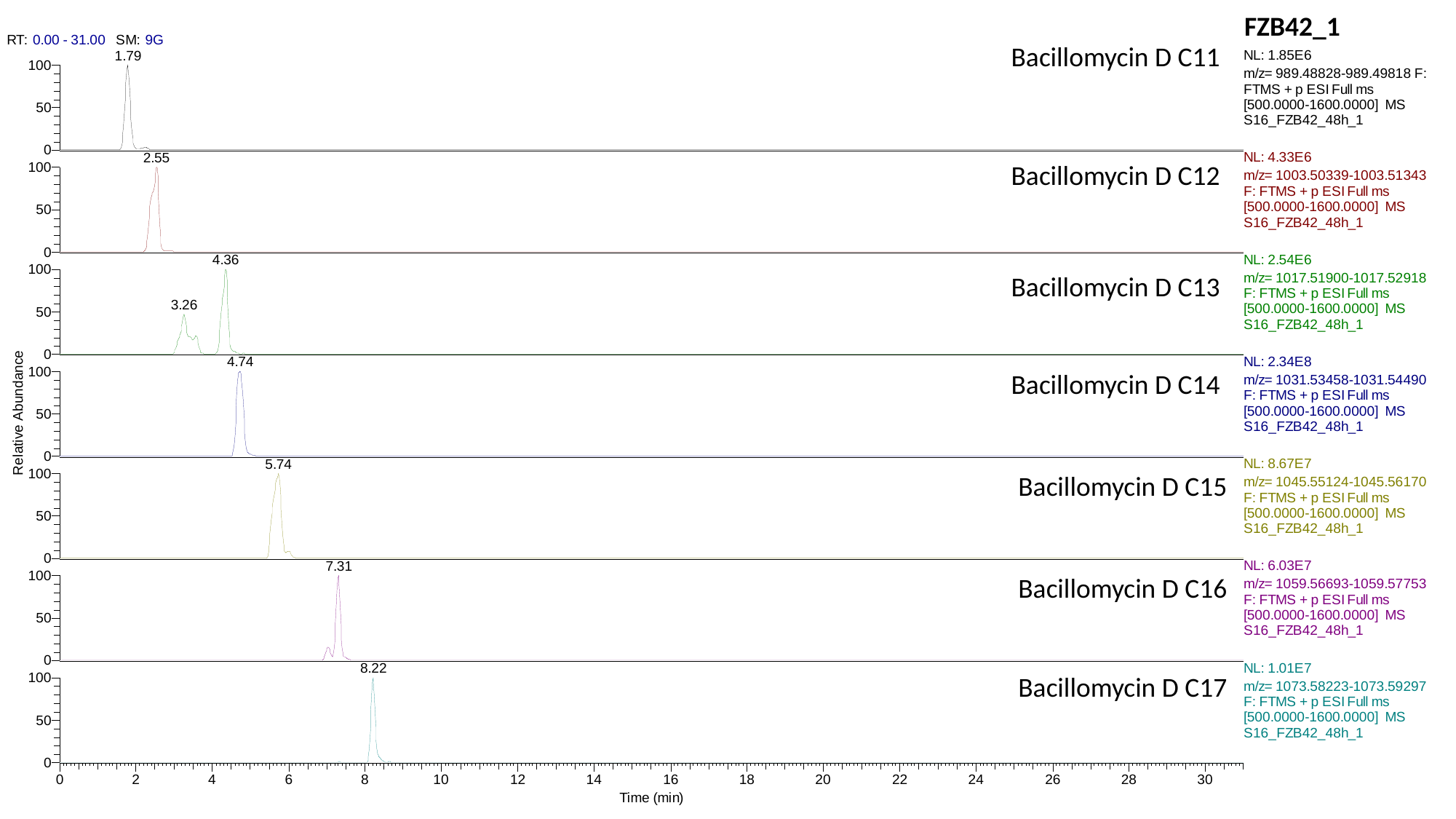

FZB42_1
Bacillomycin D C11
Bacillomycin D C12
Bacillomycin D C13
Bacillomycin D C14
Bacillomycin D C15
Bacillomycin D C16
Bacillomycin D C17

## Slide 7
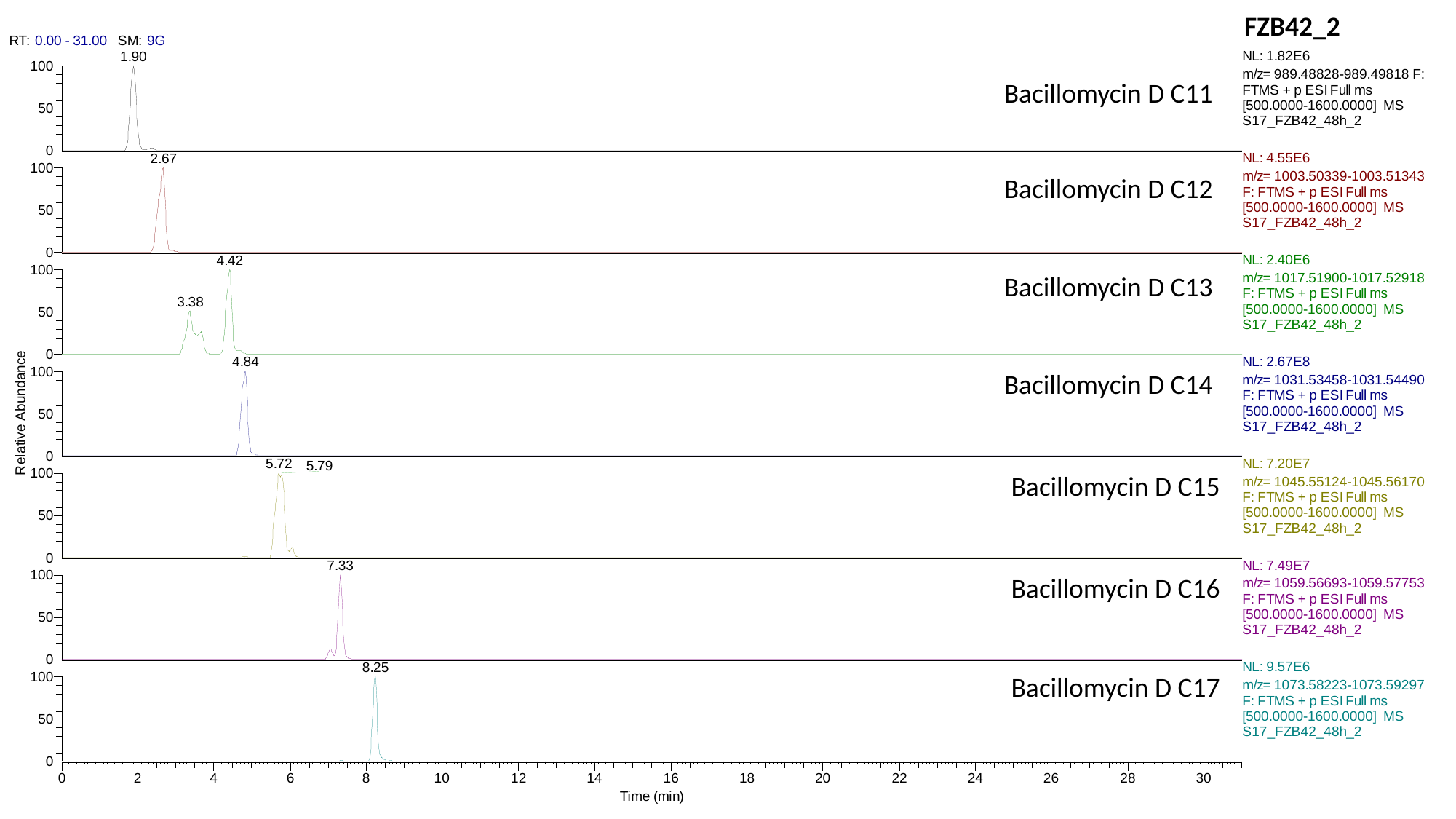

FZB42_2
Bacillomycin D C11
Bacillomycin D C12
Bacillomycin D C13
Bacillomycin D C14
Bacillomycin D C15
Bacillomycin D C16
Bacillomycin D C17

## Slide 8
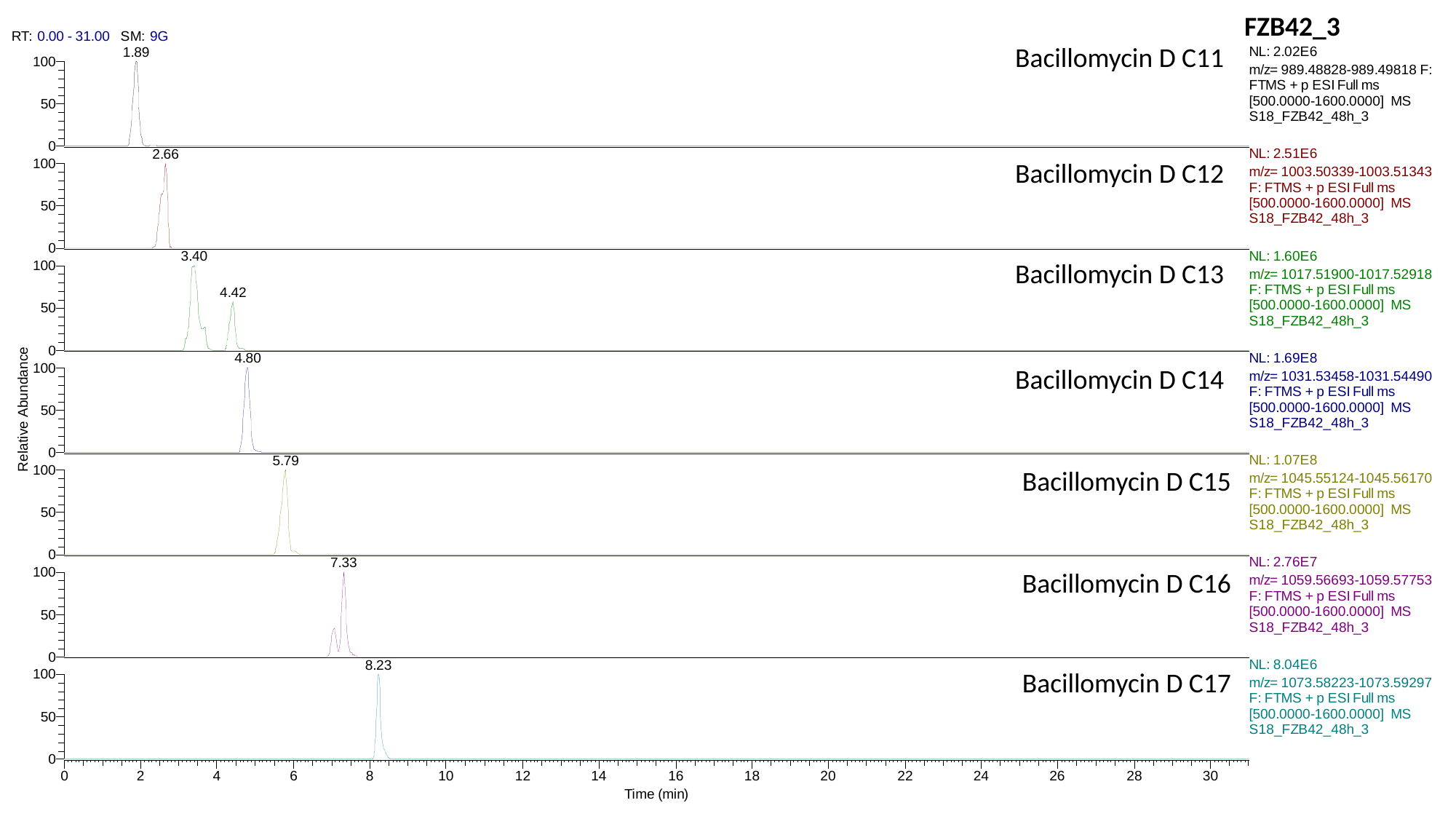

FZB42_3
Bacillomycin D C11
Bacillomycin D C12
Bacillomycin D C13
Bacillomycin D C14
Bacillomycin D C15
Bacillomycin D C16
Bacillomycin D C17

## Slide 9
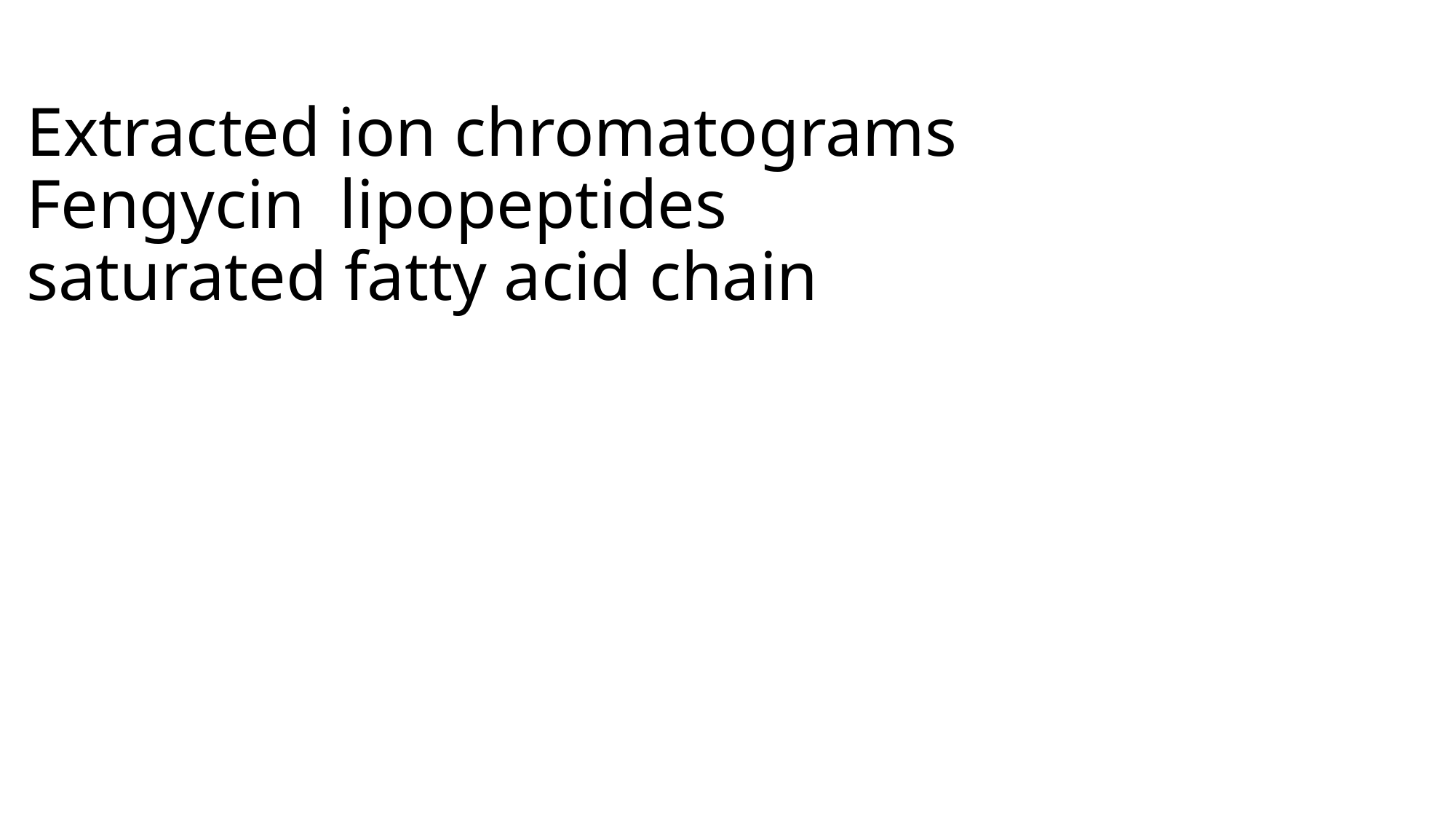

Extracted ion chromatograms
Fengycin lipopeptides
saturated fatty acid chain

## Slide 10
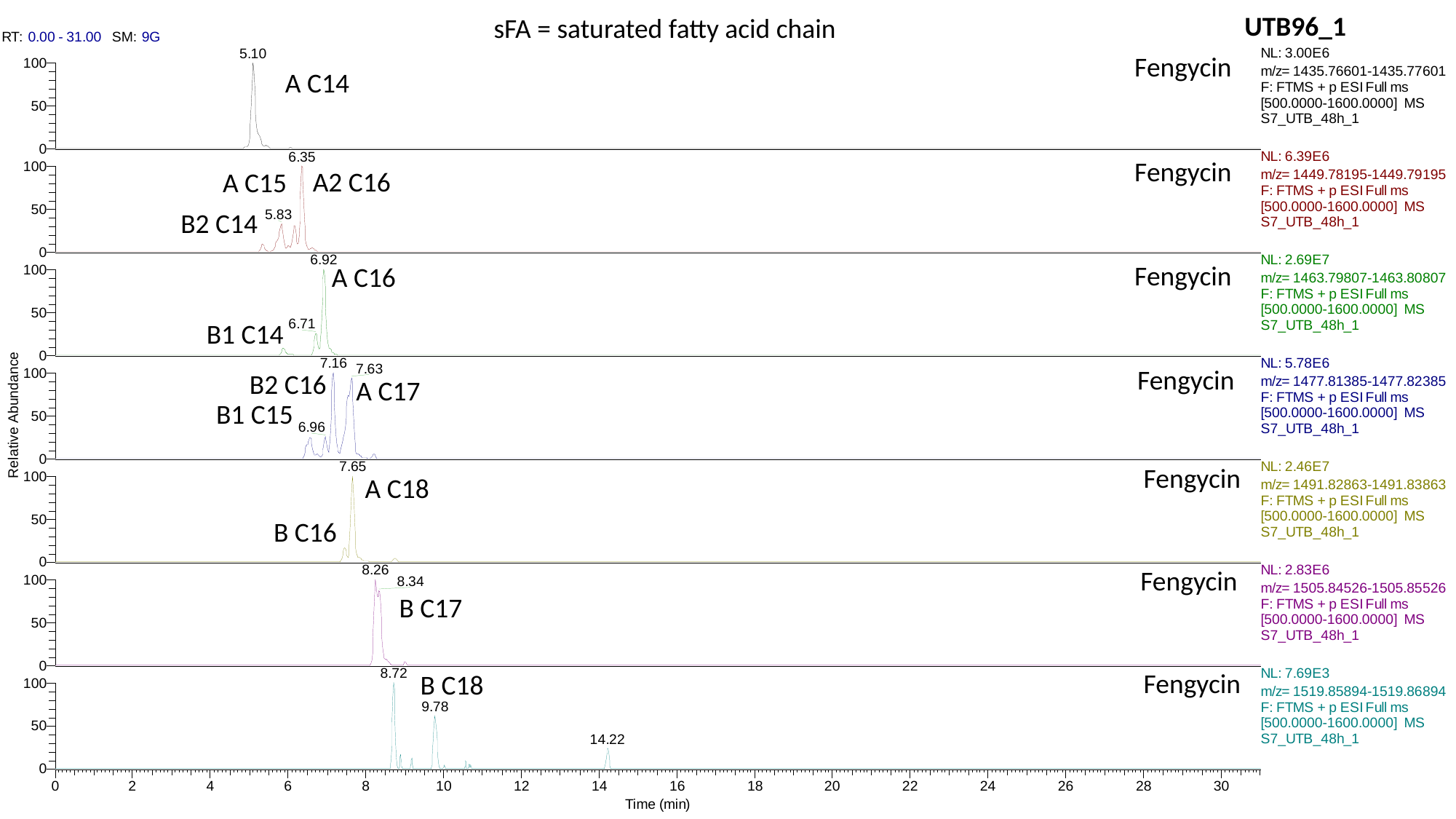

UTB96_1
sFA = saturated fatty acid chain
Fengycin
A C14
Fengycin
A2 C16
A C15
B2 C14
Fengycin
A C16
B1 C14
Fengycin
B2 C16
A C17
B1 C15
Fengycin
A C18
B C16
Fengycin
B C17
Fengycin
B C18

## Slide 11
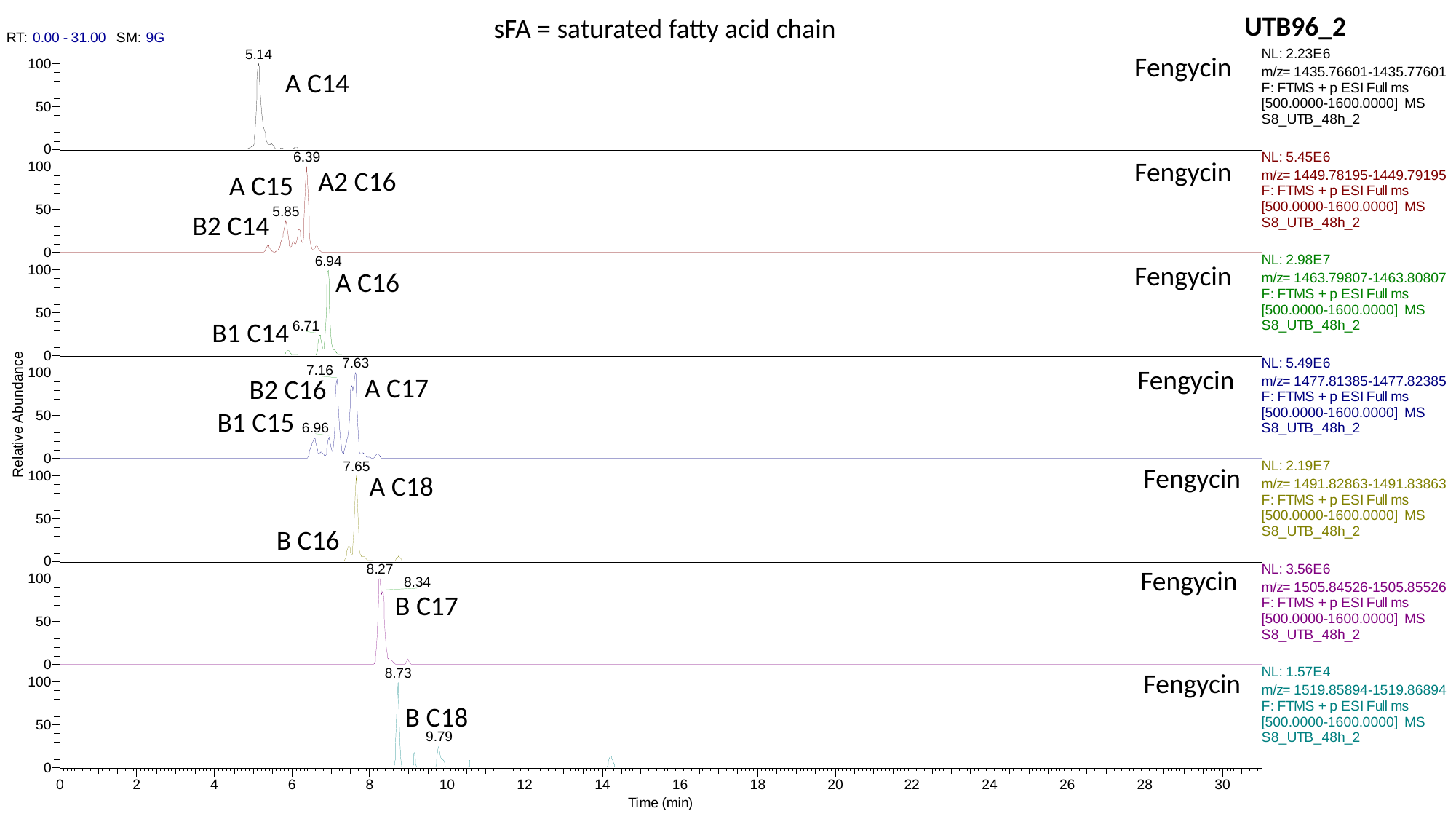

UTB96_2
sFA = saturated fatty acid chain
Fengycin
A C14
Fengycin
A2 C16
A C15
B2 C14
Fengycin
A C16
B1 C14
Fengycin
A C17
B2 C16
B1 C15
Fengycin
A C18
B C16
Fengycin
B C17
Fengycin
B C18

## Slide 12
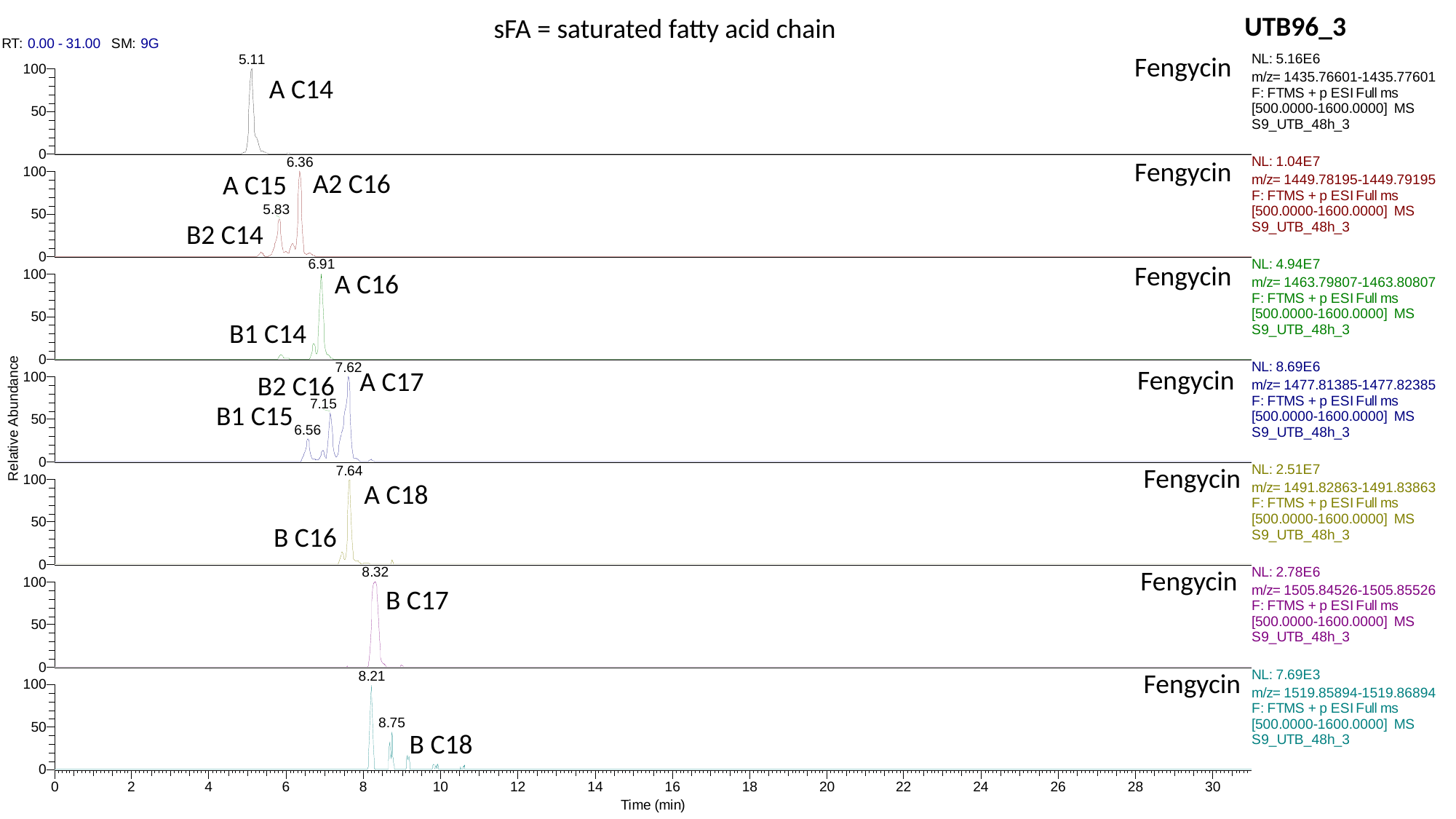

UTB96_3
sFA = saturated fatty acid chain
Fengycin
A C14
Fengycin
A2 C16
A C15
B2 C14
Fengycin
A C16
B1 C14
Fengycin
A C17
B2 C16
B1 C15
Fengycin
A C18
B C16
Fengycin
B C17
Fengycin
B C18

## Slide 13
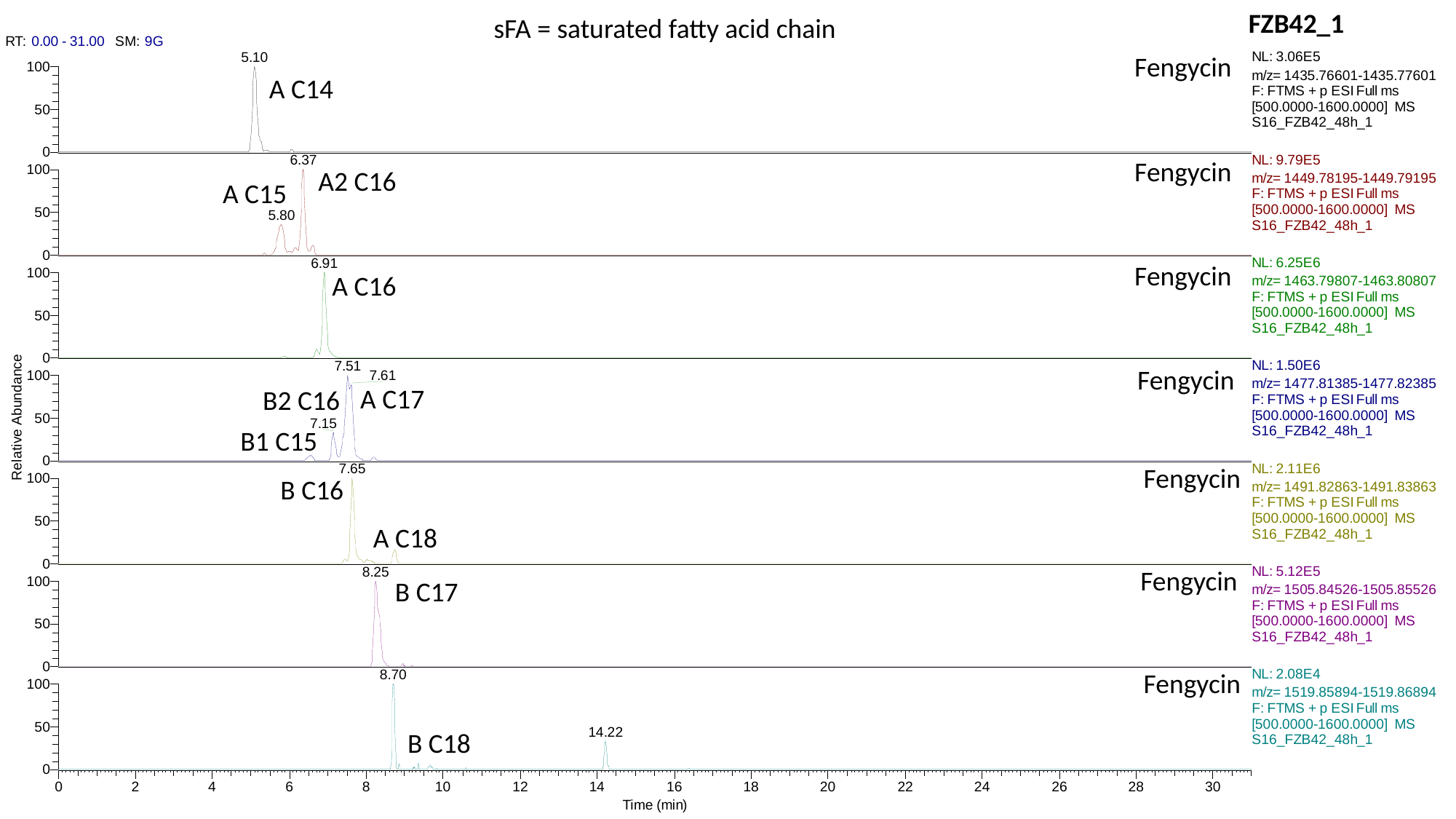

FZB42_1
sFA = saturated fatty acid chain
Fengycin
A C14
Fengycin
A2 C16
A C15
Fengycin
A C16
Fengycin
A C17
B2 C16
B1 C15
Fengycin
B C16
A C18
Fengycin
B C17
Fengycin
B C18

## Slide 14
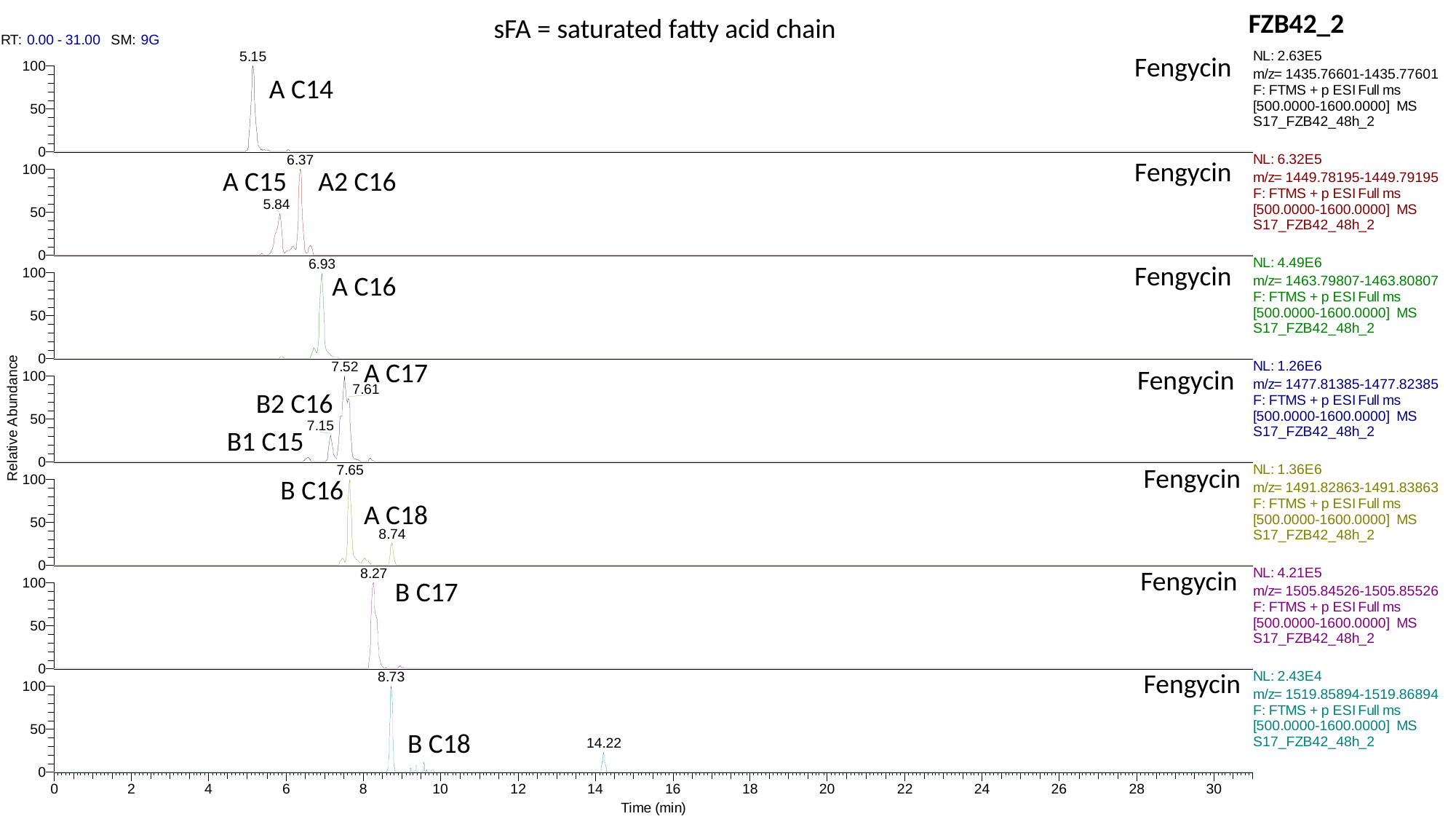

FZB42_2
sFA = saturated fatty acid chain
Fengycin
A C14
Fengycin
A C15
A2 C16
Fengycin
A C16
A C17
Fengycin
B2 C16
B1 C15
Fengycin
B C16
A C18
Fengycin
B C17
Fengycin
B C18

## Slide 15
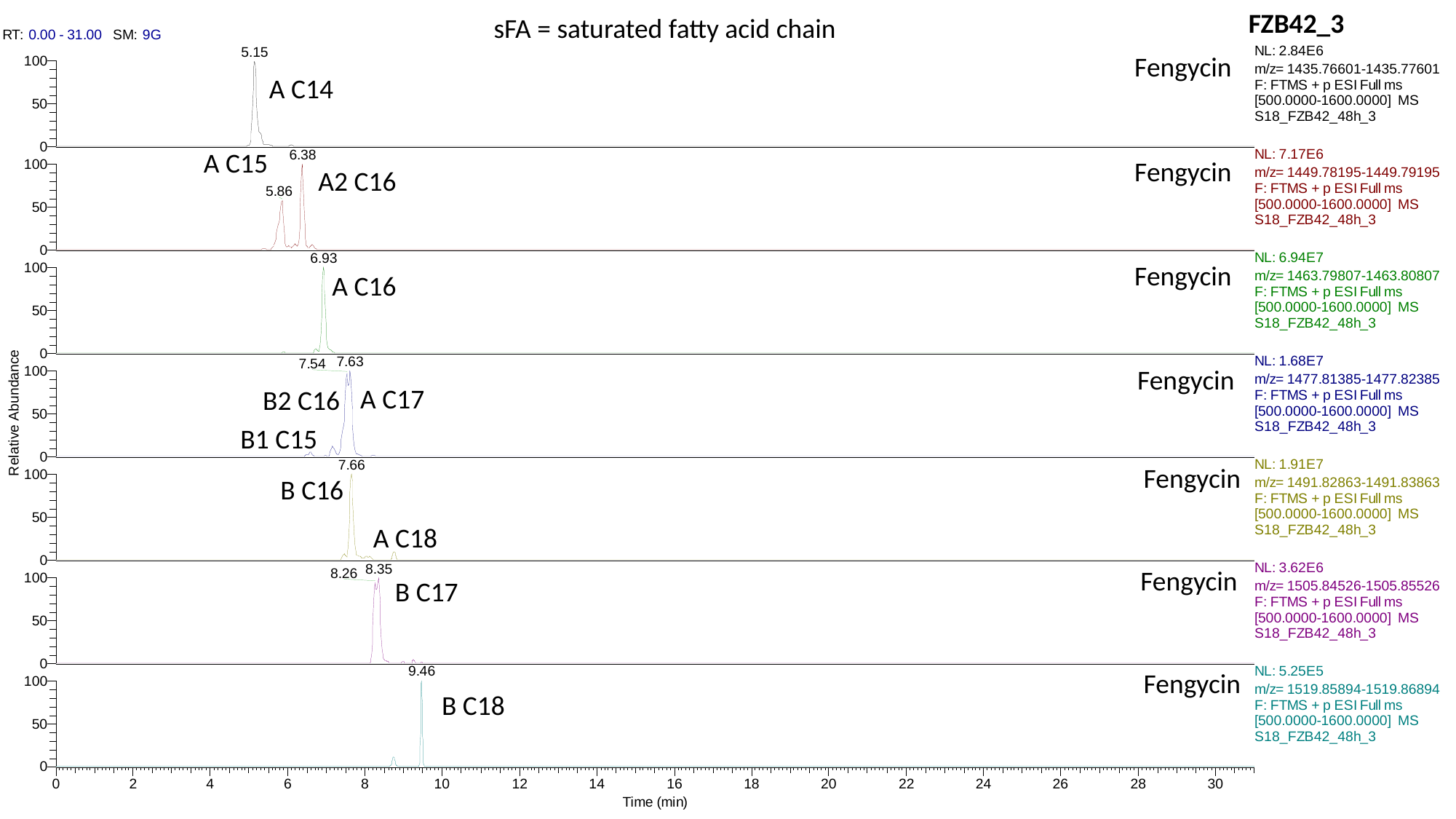

FZB42_3
sFA = saturated fatty acid chain
Fengycin
A C14
A C15
Fengycin
A2 C16
Fengycin
A C16
Fengycin
A C17
B2 C16
B1 C15
Fengycin
B C16
A C18
Fengycin
B C17
Fengycin
B C18

## Slide 16
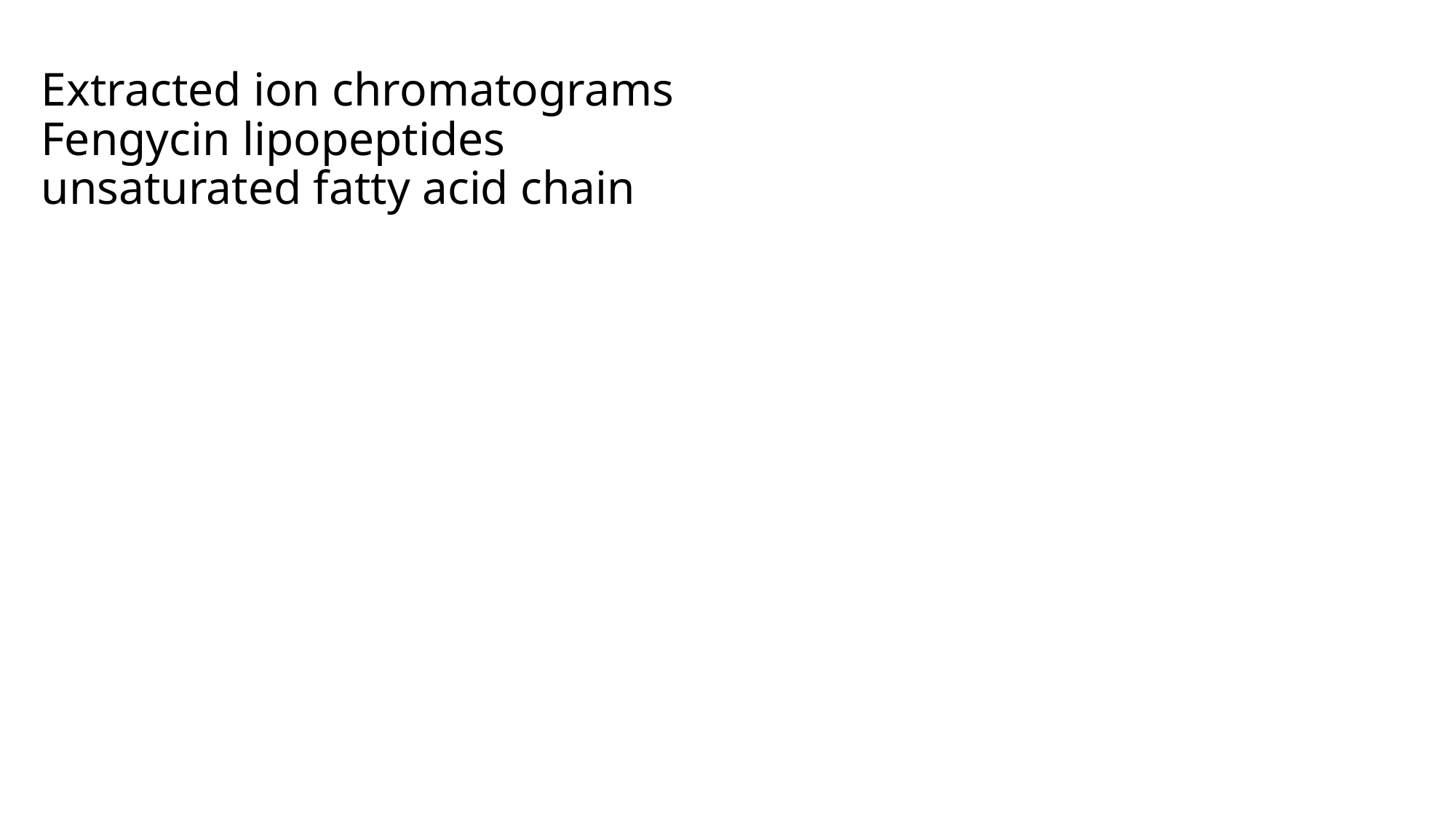

# Extracted ion chromatograms Fengycin lipopeptides unsaturated fatty acid chain

## Slide 17
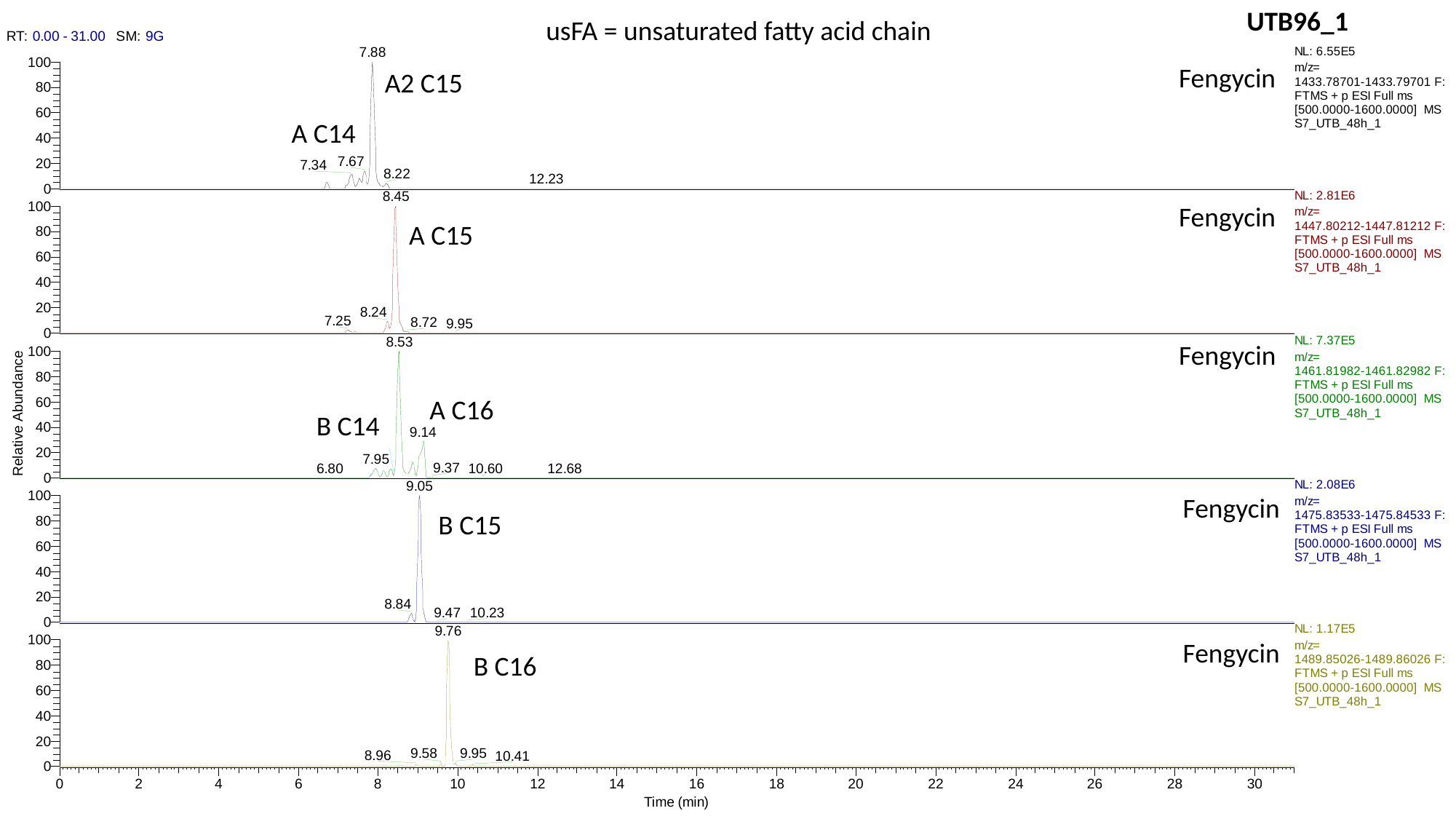

UTB96_1
usFA = unsaturated fatty acid chain
Fengycin
A2 C15
A C14
Fengycin
A C15
Fengycin
A C16
B C14
Fengycin
B C15
Fengycin
B C16

## Slide 18
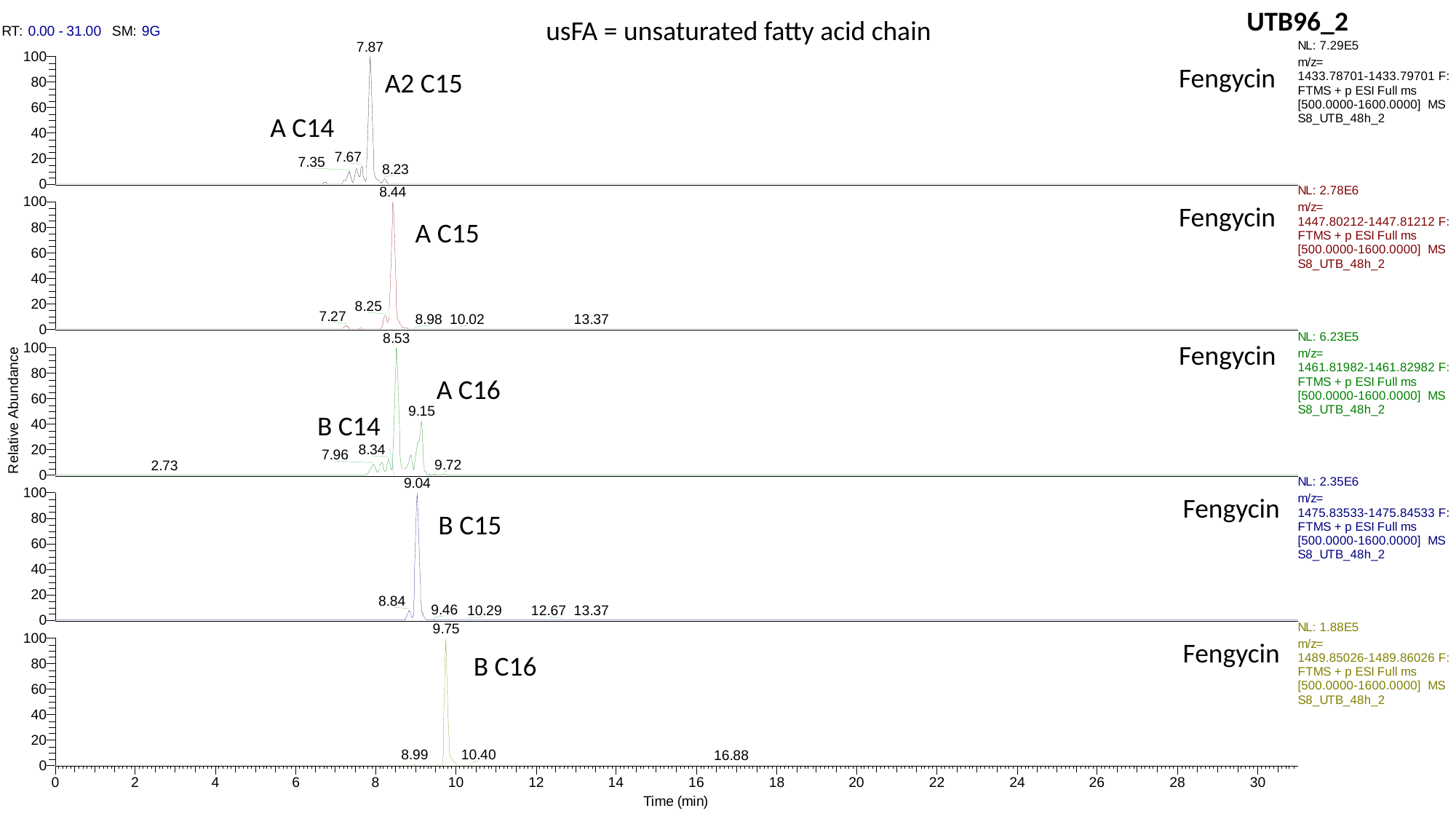

UTB96_2
usFA = unsaturated fatty acid chain
Fengycin
A2 C15
A C14
Fengycin
A C15
Fengycin
A C16
B C14
Fengycin
B C15
Fengycin
B C16

## Slide 19
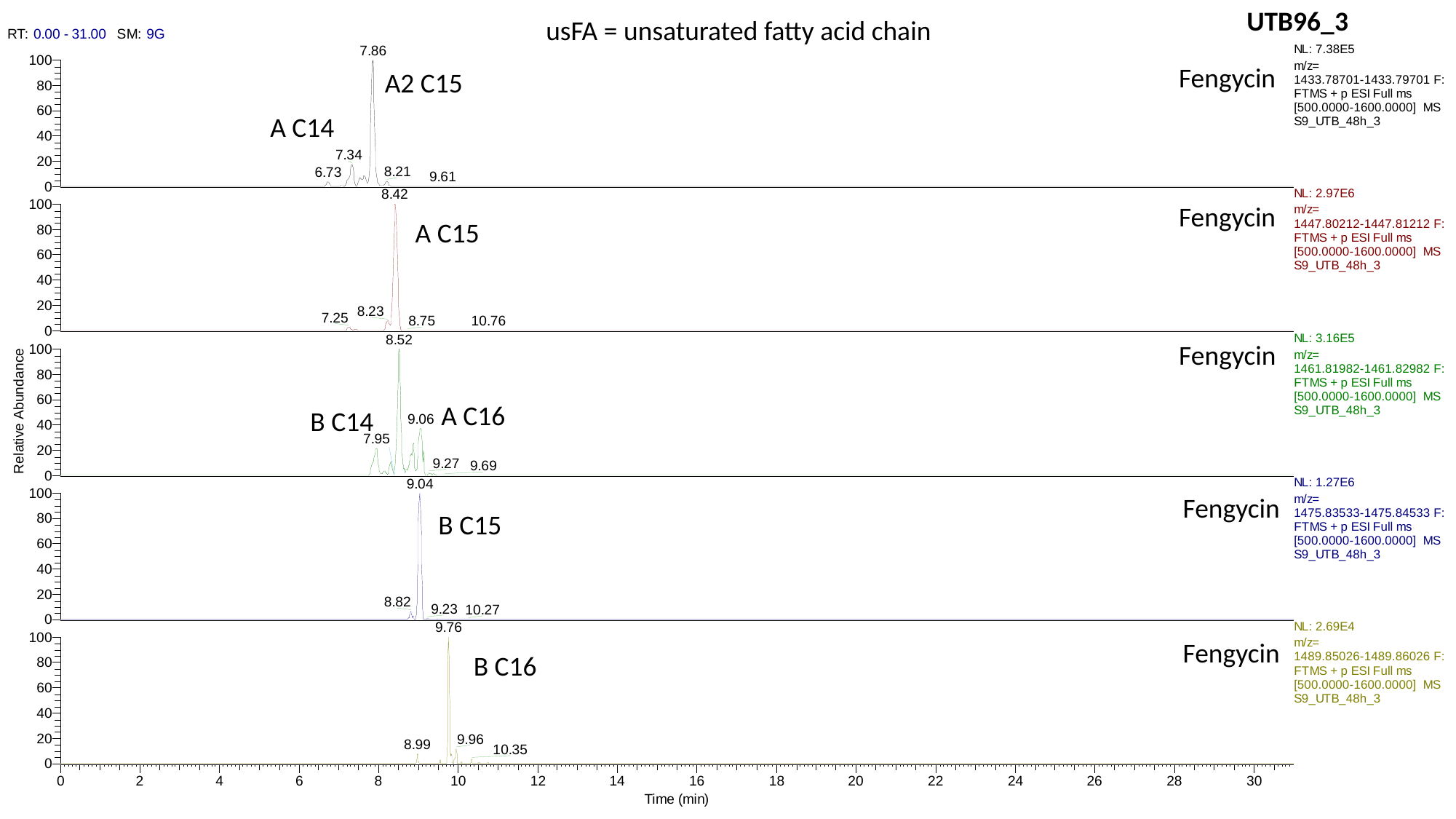

UTB96_3
usFA = unsaturated fatty acid chain
Fengycin
A2 C15
A C14
Fengycin
A C15
Fengycin
A C16
B C14
Fengycin
B C15
Fengycin
B C16

## Slide 20
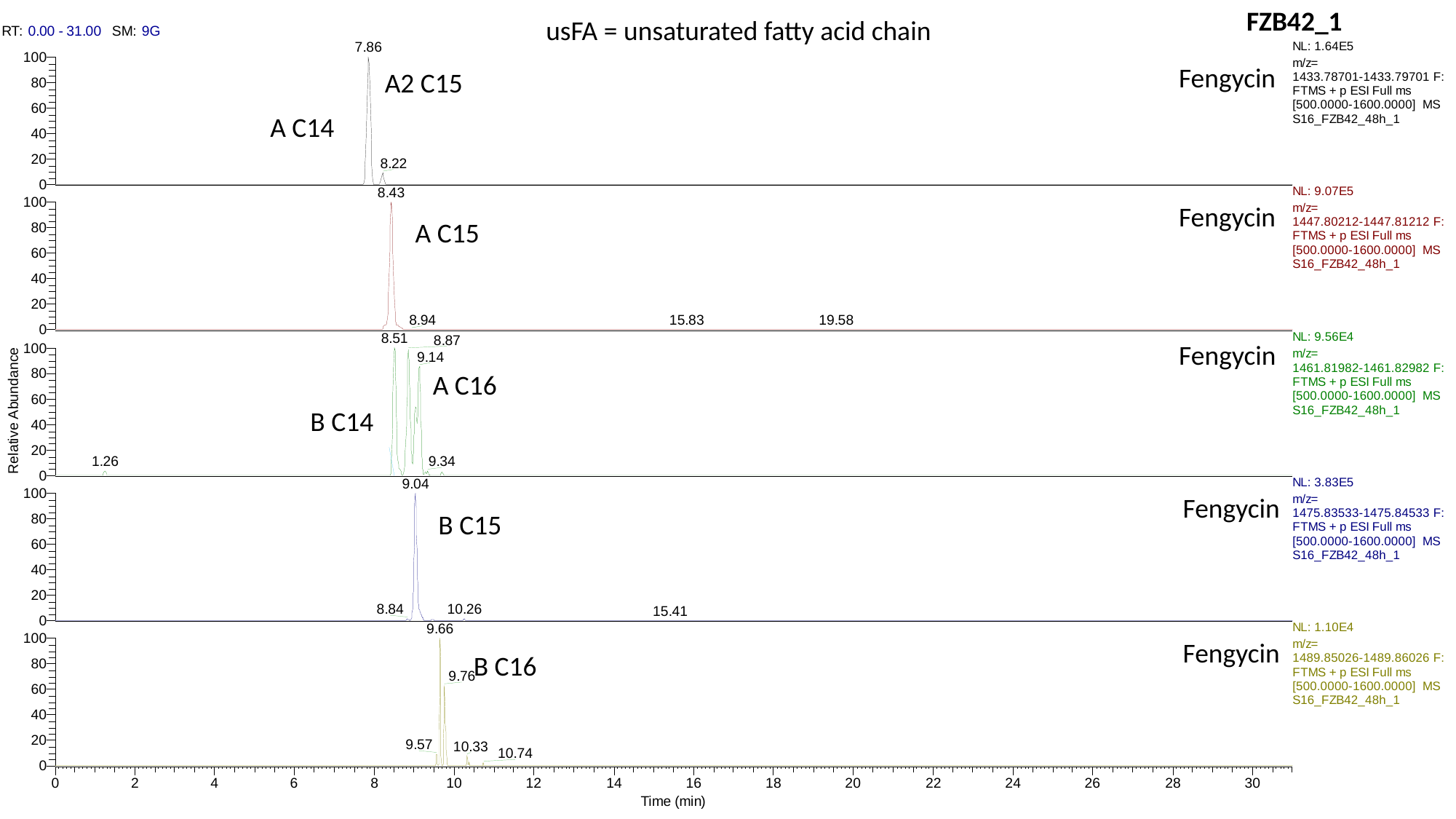

FZB42_1
usFA = unsaturated fatty acid chain
Fengycin
A2 C15
A C14
Fengycin
A C15
Fengycin
A C16
B C14
Fengycin
B C15
Fengycin
B C16

## Slide 21
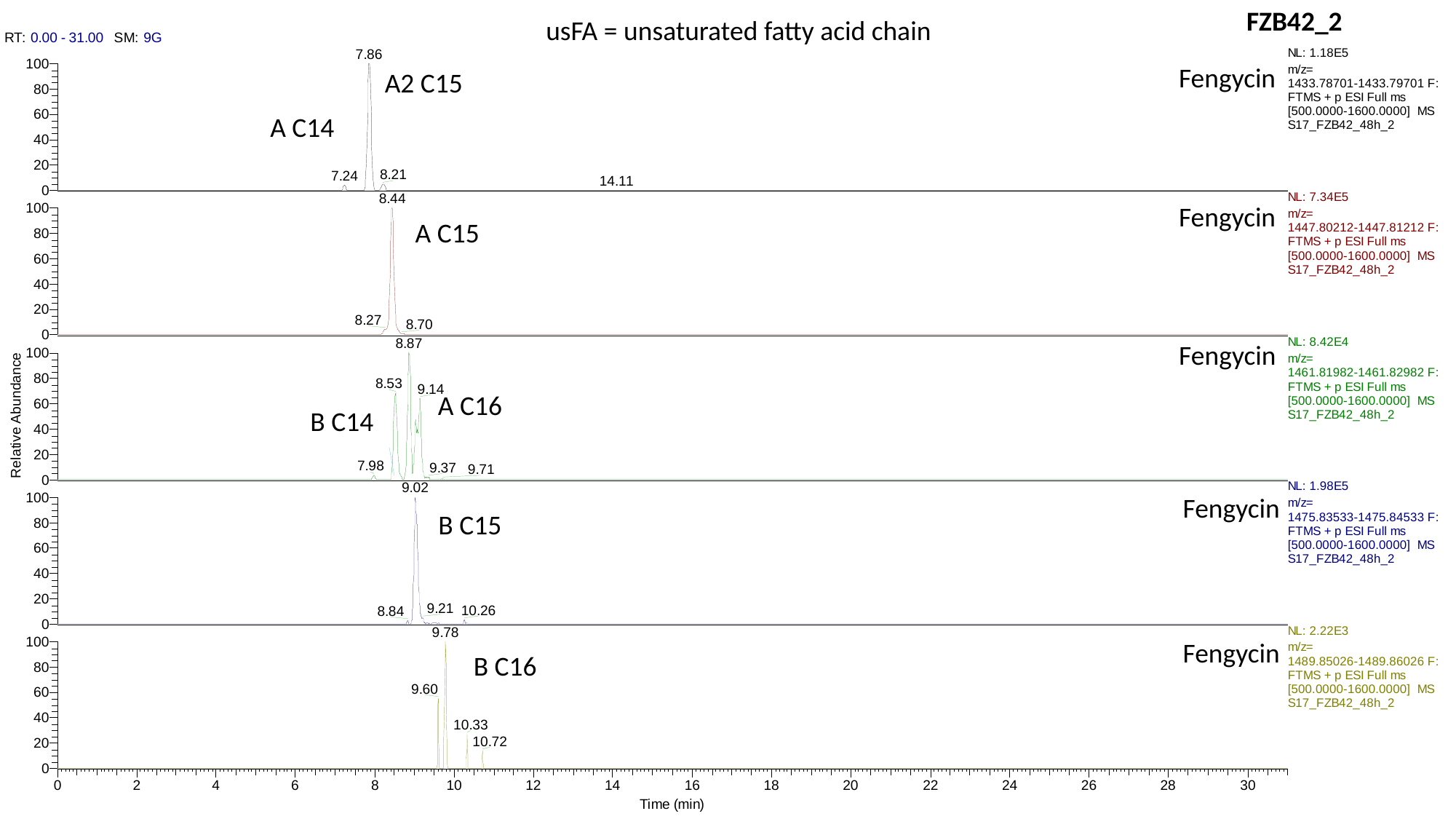

FZB42_2
usFA = unsaturated fatty acid chain
Fengycin
A2 C15
A C14
Fengycin
A C15
Fengycin
A C16
B C14
Fengycin
B C15
Fengycin
B C16

## Slide 22
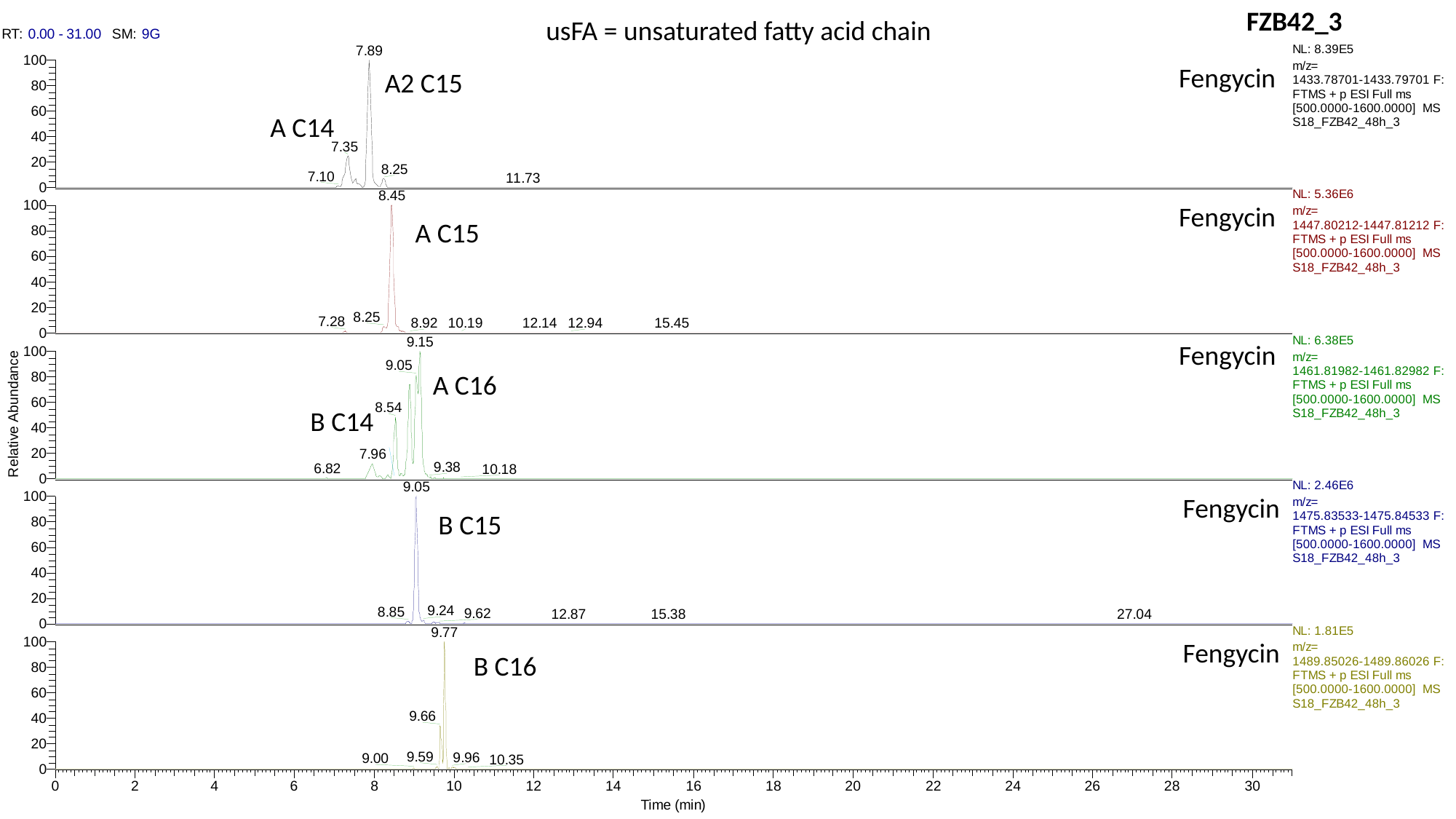

FZB42_3
usFA = unsaturated fatty acid chain
Fengycin
A2 C15
A C14
Fengycin
A C15
Fengycin
A C16
B C14
Fengycin
B C15
Fengycin
B C16

## Slide 23
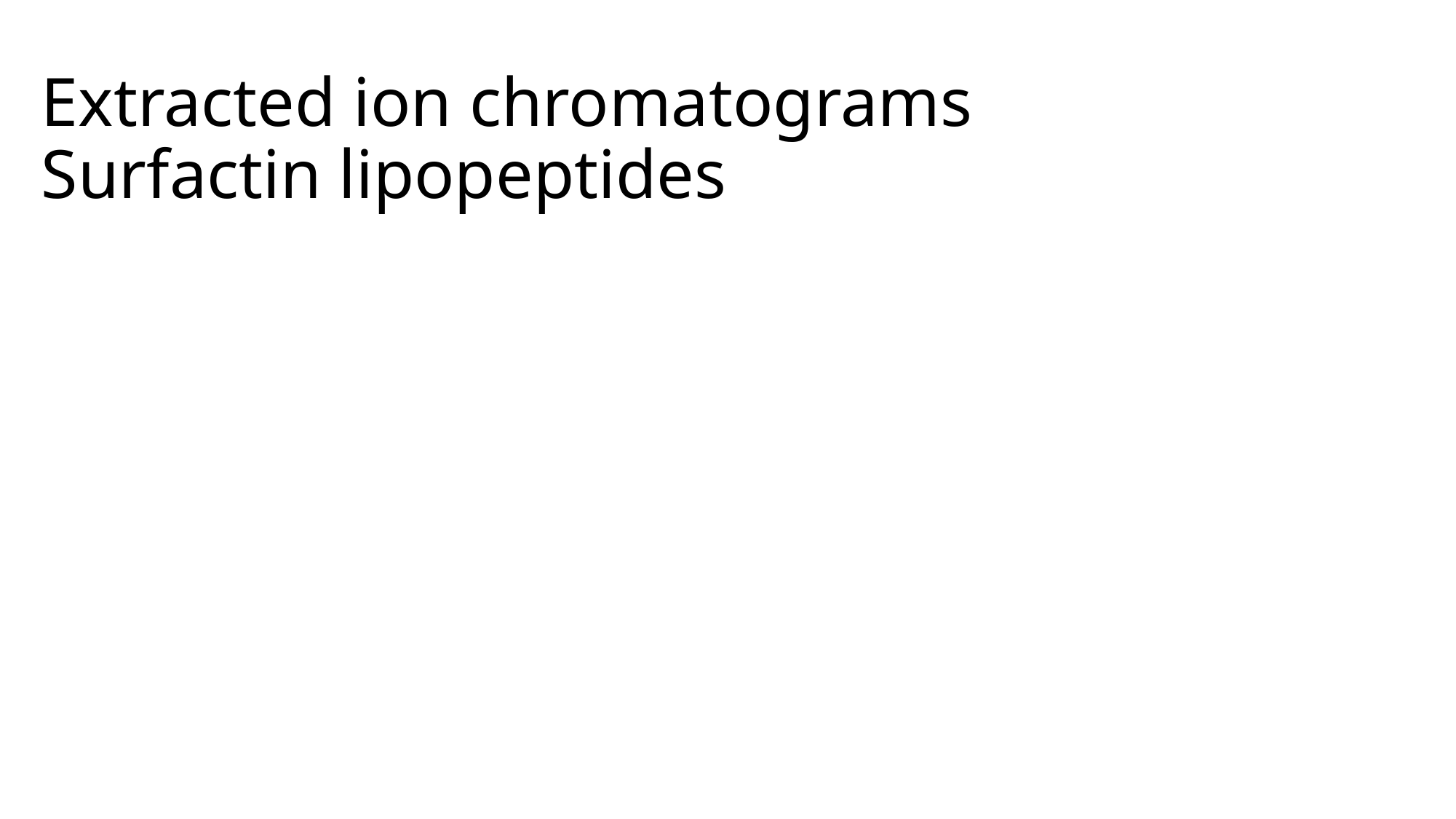

# Extracted ion chromatograms Surfactin lipopeptides

## Slide 24
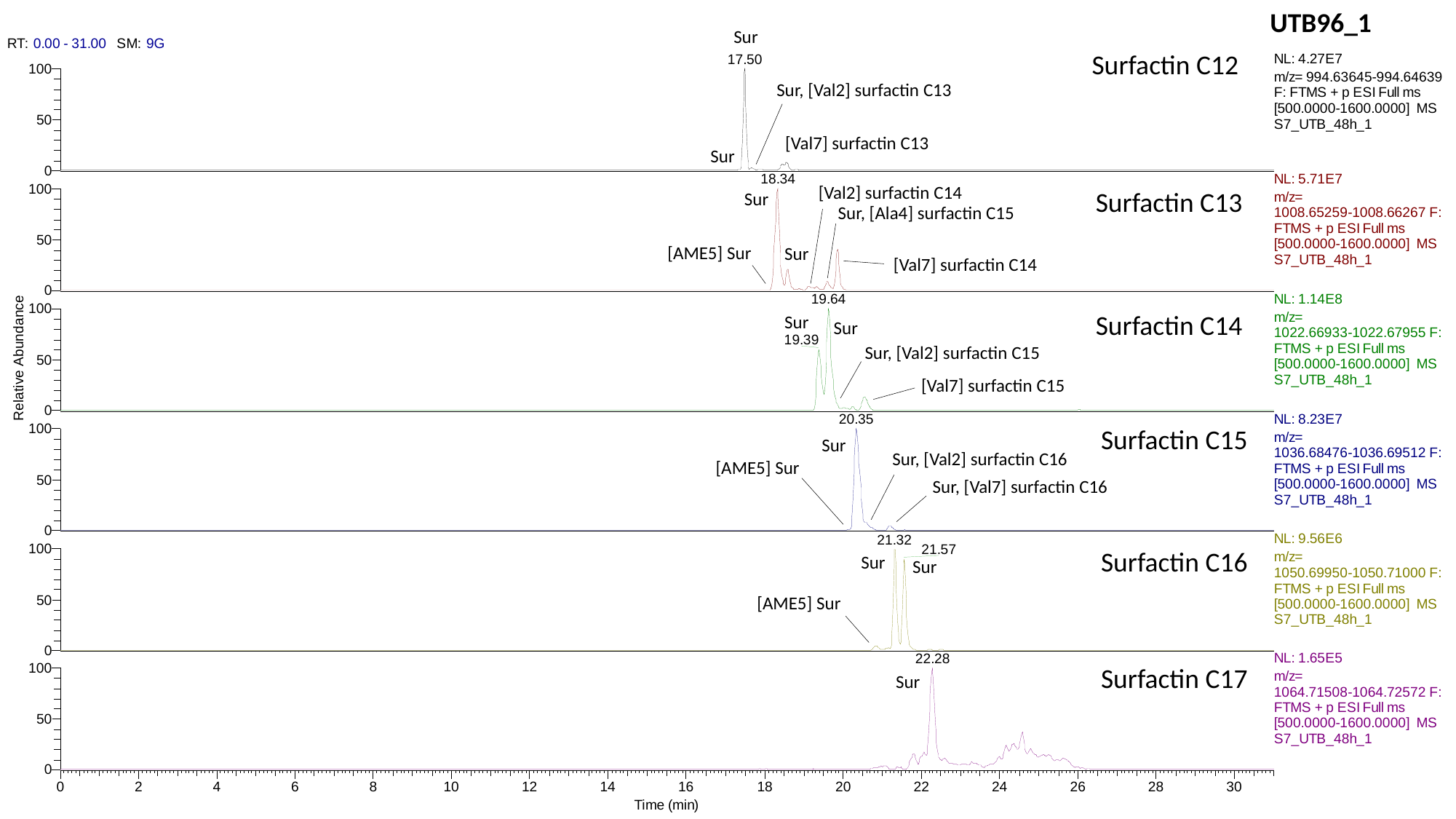

UTB96_1
Sur
Surfactin C12
Sur, [Val2] surfactin C13
[Val7] surfactin C13
Sur
[Val2] surfactin C14
Surfactin C13
Sur
Sur, [Ala4] surfactin C15
[AME5] Sur
Sur
[Val7] surfactin C14
Surfactin C14
Sur
Sur
Sur, [Val2] surfactin C15
[Val7] surfactin C15
Surfactin C15
Sur
Sur, [Val2] surfactin C16
[AME5] Sur
Sur, [Val7] surfactin C16
Surfactin C16
Sur
Sur
[AME5] Sur
Surfactin C17
Sur

## Slide 25
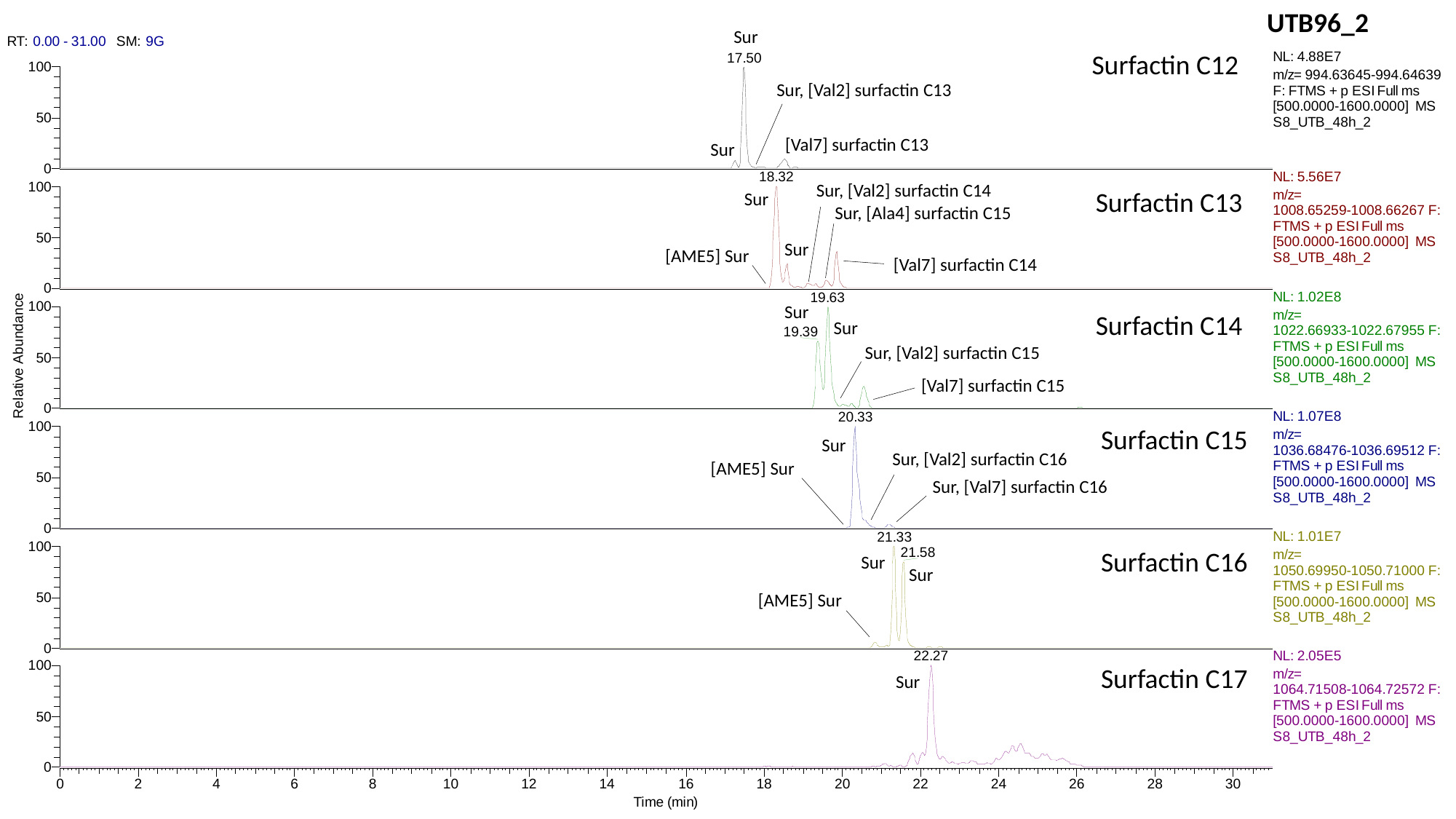

UTB96_2
Sur
Surfactin C12
Sur, [Val2] surfactin C13
[Val7] surfactin C13
Sur
Sur, [Val2] surfactin C14
Surfactin C13
Sur
Sur, [Ala4] surfactin C15
Sur
[AME5] Sur
[Val7] surfactin C14
Sur
Surfactin C14
Sur
Sur, [Val2] surfactin C15
[Val7] surfactin C15
Surfactin C15
Sur
Sur, [Val2] surfactin C16
[AME5] Sur
Sur, [Val7] surfactin C16
Surfactin C16
Sur
Sur
[AME5] Sur
Surfactin C17
Sur

## Slide 26
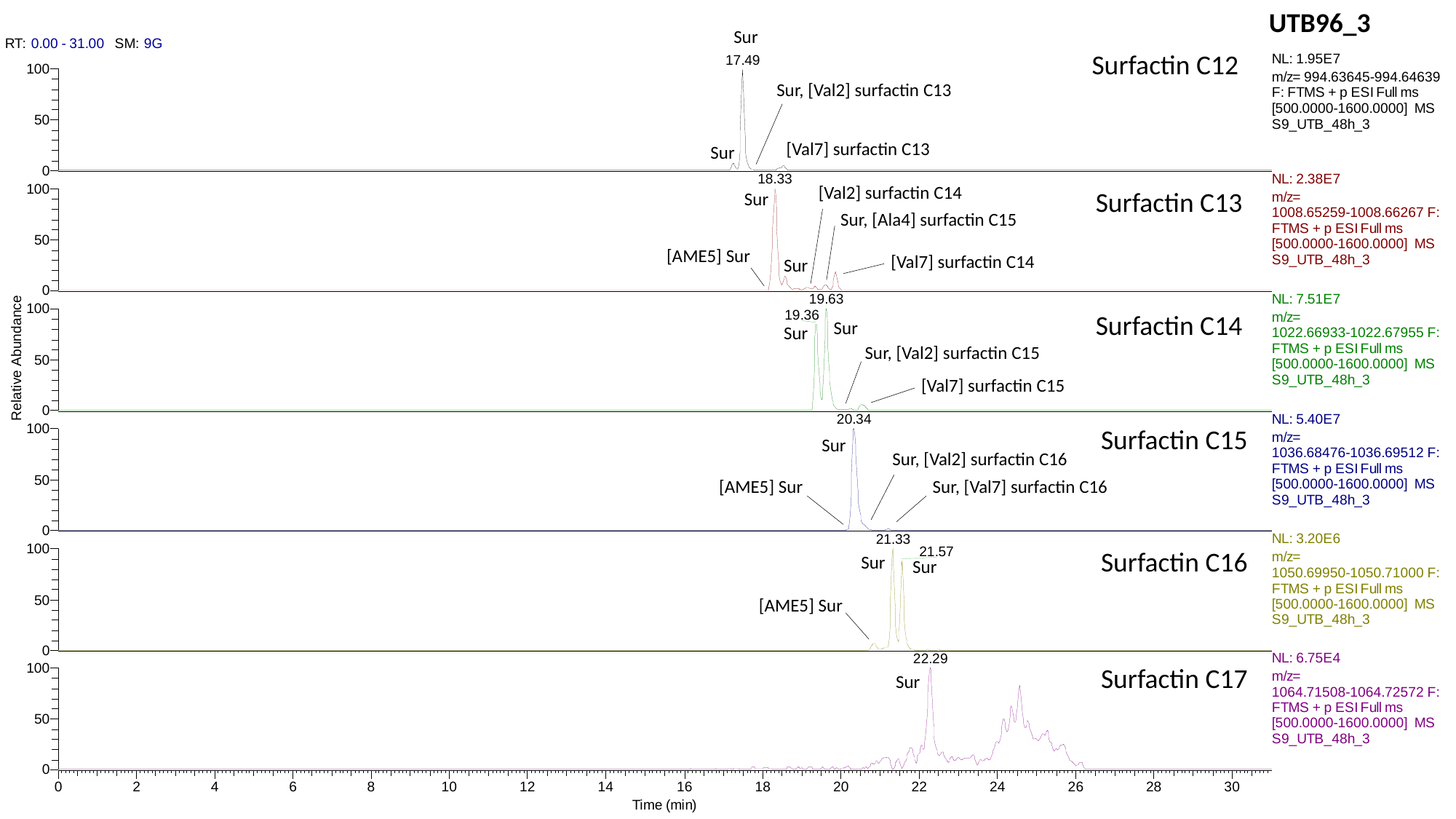

UTB96_3
Sur
Surfactin C12
Sur, [Val2] surfactin C13
[Val7] surfactin C13
Sur
[Val2] surfactin C14
Surfactin C13
Sur
Sur, [Ala4] surfactin C15
[AME5] Sur
[Val7] surfactin C14
Sur
Surfactin C14
Sur
Sur
Sur, [Val2] surfactin C15
[Val7] surfactin C15
Surfactin C15
Sur
Sur, [Val2] surfactin C16
[AME5] Sur
Sur, [Val7] surfactin C16
Surfactin C16
Sur
Sur
[AME5] Sur
Surfactin C17
Sur

## Slide 27
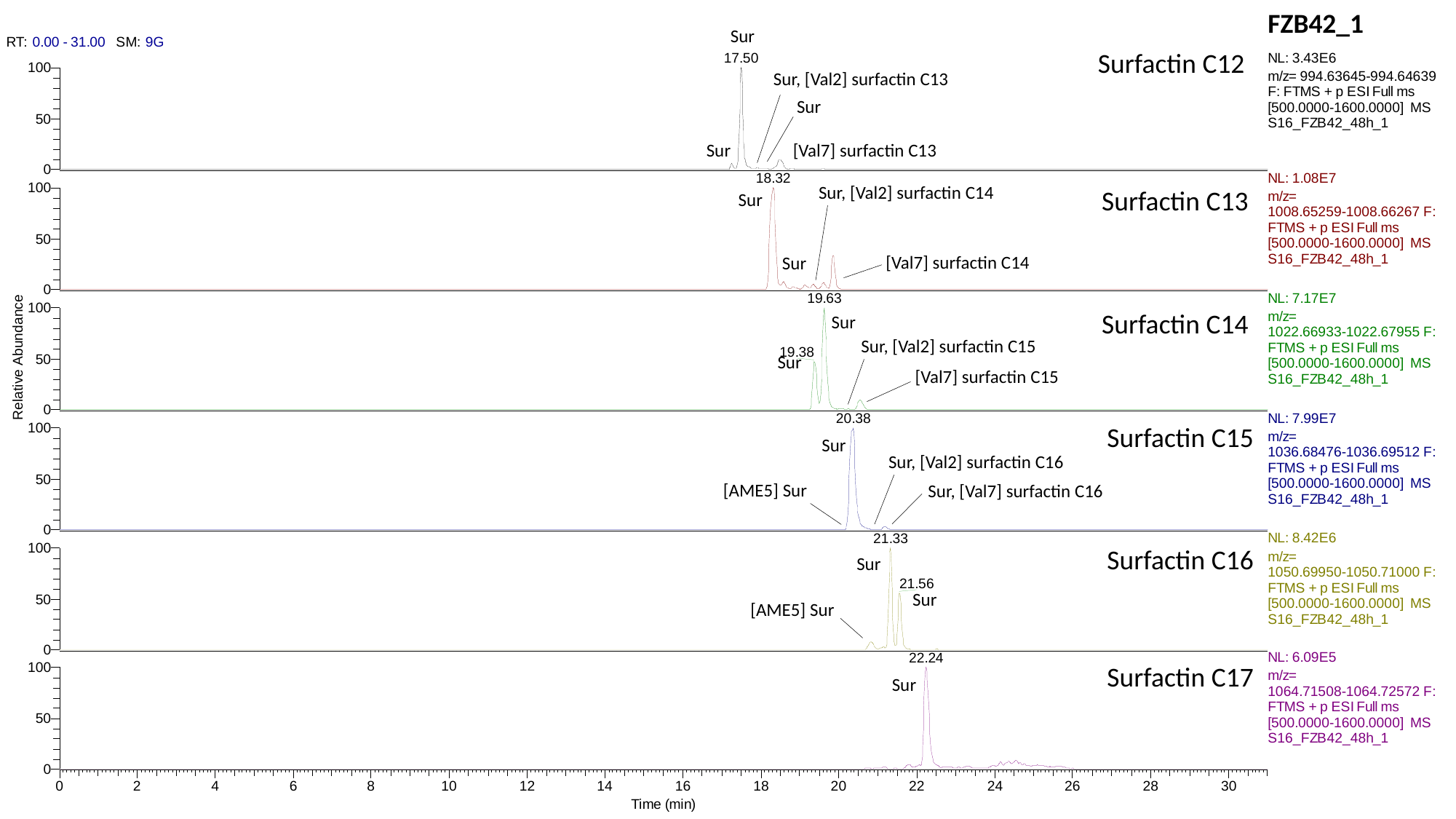

FZB42_1
Sur
Surfactin C12
Sur, [Val2] surfactin C13
Sur
[Val7] surfactin C13
Sur
Sur, [Val2] surfactin C14
Surfactin C13
Sur
[Val7] surfactin C14
Sur
Surfactin C14
Sur
Sur, [Val2] surfactin C15
Sur
[Val7] surfactin C15
Surfactin C15
Sur
Sur, [Val2] surfactin C16
[AME5] Sur
Sur, [Val7] surfactin C16
Surfactin C16
Sur
Sur
[AME5] Sur
Surfactin C17
Sur

## Slide 28
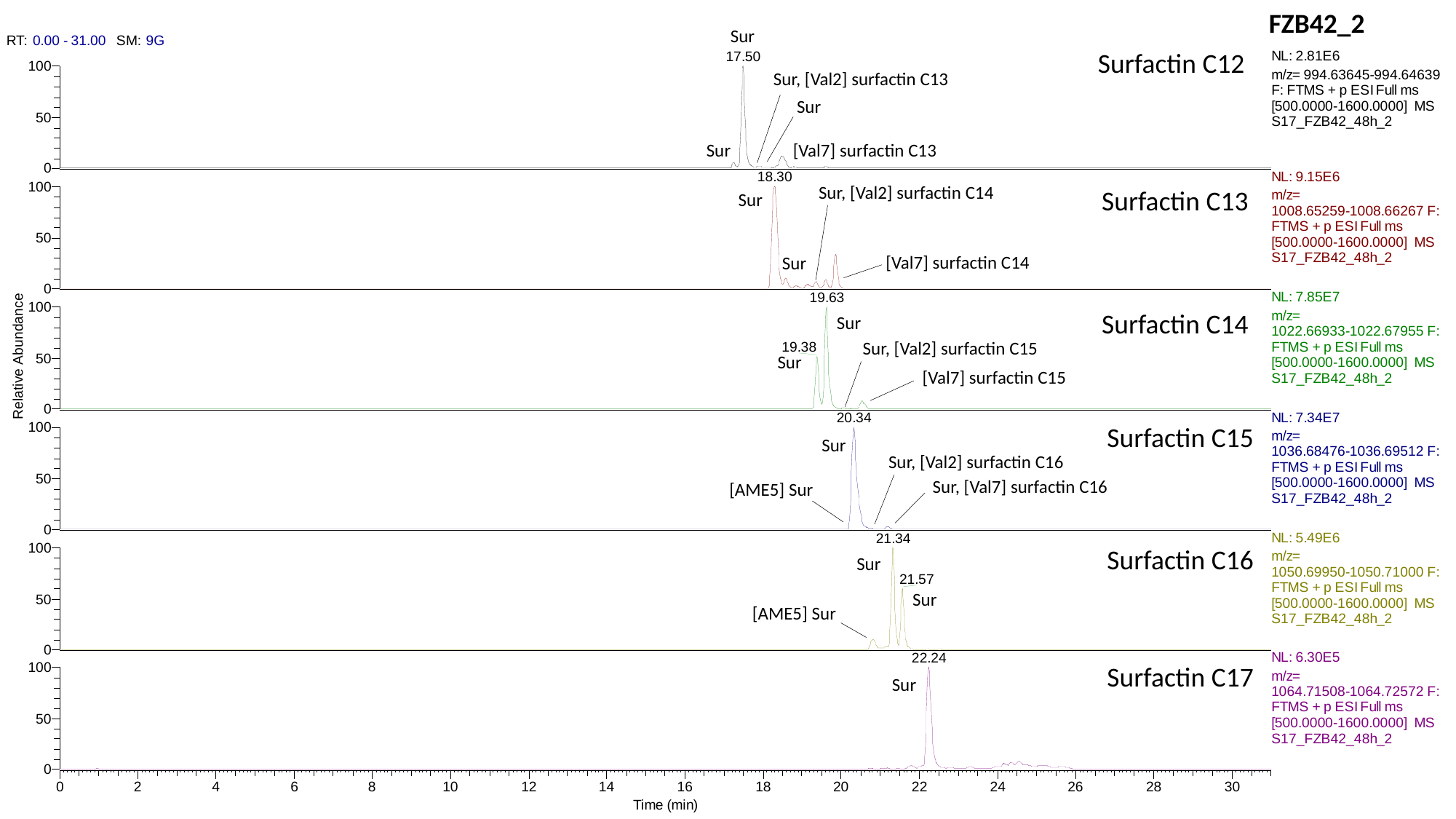

FZB42_2
Sur
Surfactin C12
Sur, [Val2] surfactin C13
Sur
[Val7] surfactin C13
Sur
Sur, [Val2] surfactin C14
Surfactin C13
Sur
[Val7] surfactin C14
Sur
Surfactin C14
Sur
Sur, [Val2] surfactin C15
Sur
[Val7] surfactin C15
Surfactin C15
Sur
Sur, [Val2] surfactin C16
Sur, [Val7] surfactin C16
[AME5] Sur
Surfactin C16
Sur
Sur
[AME5] Sur
Surfactin C17
Sur

## Slide 29
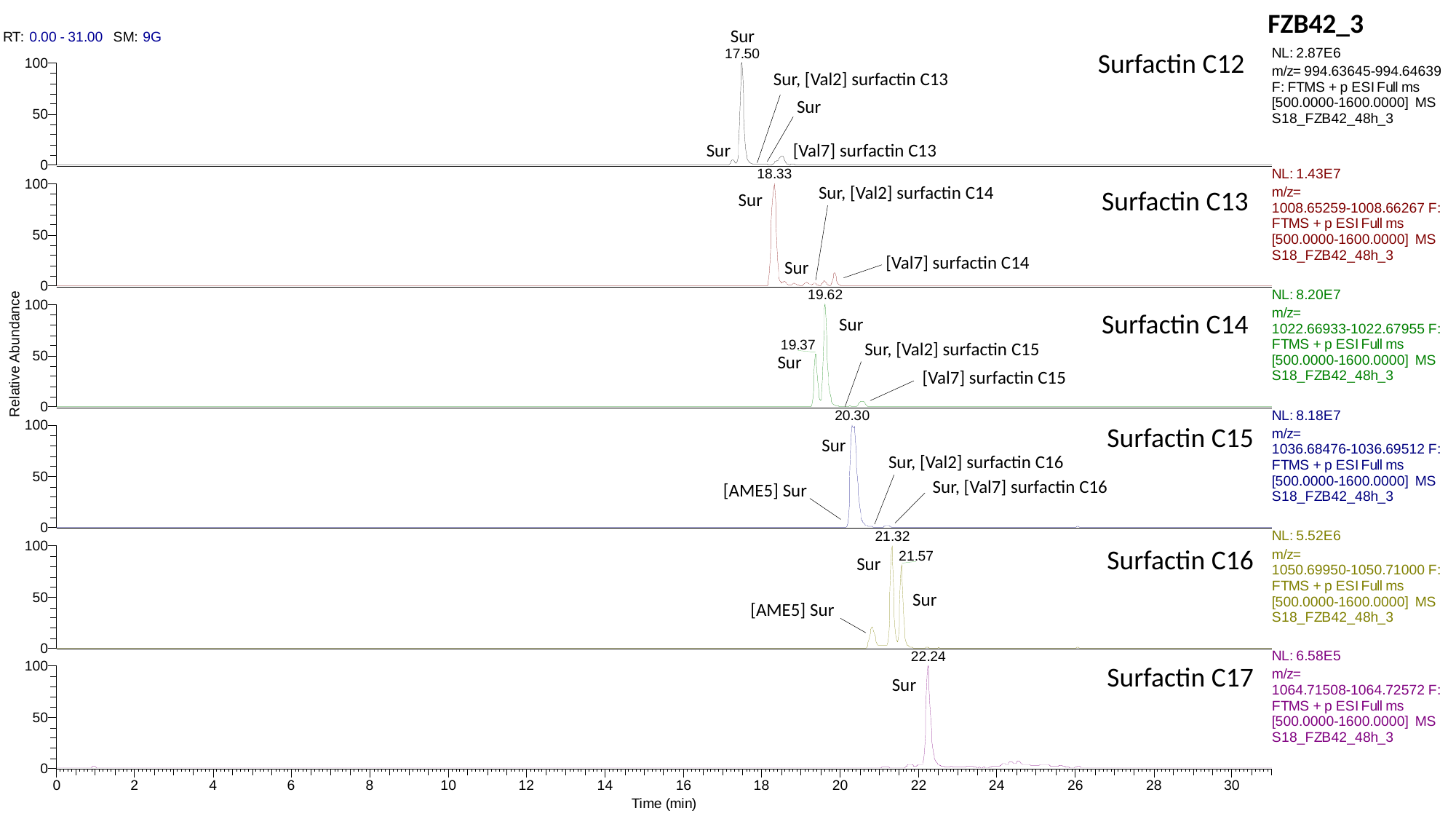

FZB42_3
Sur
Surfactin C12
Sur, [Val2] surfactin C13
Sur
[Val7] surfactin C13
Sur
Sur, [Val2] surfactin C14
Surfactin C13
Sur
[Val7] surfactin C14
Sur
Surfactin C14
Sur
Sur, [Val2] surfactin C15
Sur
[Val7] surfactin C15
Surfactin C15
Sur
Sur, [Val2] surfactin C16
Sur, [Val7] surfactin C16
[AME5] Sur
Surfactin C16
Sur
Sur
[AME5] Sur
Surfactin C17
Sur
